# Homology Modeling Study of Bovine μ-Calpain Inhibitor-Binding Domains

**DOI:** 10.3390/ijms15057897

**Published:** 2014-05-06

**Authors:** Han-Ha Chai, Dajeong Lim, Seung-Hwan Lee, Hee-Yeoul Chai, Eunkyoung Jung

**Affiliations:** 1 Animal Genome & Bioinformatics Division, National Institute of Animal Science, RDA, Suwon 441-706, Korea; E-Mail: lim.dj@korea.kr; 2 Hanwoo Experiment Station, National Institute of Animal Science, RDA, PyeongChang 232-950, Korea; E-Mail: slee46@korea.kr; 3 Division of Biosafety Evaluation and Control, Korea National Institute of Helth 187 Osongsaengmyeong2-ro, Gango-myeon, Cheongwon-gun, Chungcheongbuk-do 363-951, Korea; E-Mail: fmdv98@hanmail.net; 4 Insilicotech Co., Ltd., C-602 Korea Bio Park, 694-1 Sampyeong-Dong, Bundang-Gu, Seongnam-Shi, Gyeonggi-do 463-400, Korea; E-Mail: jungek@insilico.co.kr

**Keywords:** CAPN (Calpain), CAST4 (Calpain 4 domain), homology modeling, protease regulation system

## Abstract

The activated mammalian CAPN-structures, the CAPN/CAST complex in particular, have become an invaluable target model using the structure-based virtual screening of drug candidates from the discovery phase to development for over-activated CAPN linked to several diseases, such as post-ischemic injury and cataract formation. The effect of Ca^2+^-binding to the enzyme is thought to include activation, as well as the dissociation, aggregation, and autolysis of small regular subunits. Unfortunately, the Ca^2+^-activated enzyme tends to aggregate when provided as a divalent ion at the high-concentration required for the protease crystallization. This is also makes it very difficult to crystallize the whole-length enzyme itself, as well as the enzyme-inhibitor complex. Several parameters that influence CAPN activity have been investigated to determine its roles in Ca^2+^-modulation, autoproteolysis, phosphorylation, and intracellular distribution and inhibition by its endogenous inhibitor CAST. CAST binds and inhibits CAPN via its CAPN-inhibitor domains (four repeating domains 1–4; CAST1–4) when CAPN is activated by Ca^2+^-binding. An important key to understanding CAPN1 inhibition by CAST is to determine how CAST interacts at the molecular level with CAPN1 to inhibit its protease activity. In this study, a 3D structure model of a CAPN1 bound bovine CAST4 complex was built by comparative modeling based on the only known template structure of a rat CAPN2/CAST4 complex. The complex model suggests certain residues of bovine CAST4, notably, the TIPPKYQ motif sequence, and the structural elements of these residues, which are important for CAPN1 inhibition. In particular, as CAST4 docks near the flexible active site of CAPN1, conformational changes at the interaction site after binding could be directly related to CAST4 inhibitory activity. These functional interfaces can serve as a guide to the site-mutagenesis in research on bovine CAPN1 structure-function relationships for the design of small molecules inhibitors to prevent uncontrolled and unspecific degradation in the proteolysis of key protease substrates.

## 1. Introduction

Calpains are a family of intracellular calcium-dependent, cysteine proteases that are known in 15 mammalian isoforms and exist in almost all eukaryotes. They are classified by two criteria: their domain architectures and their patterns of expression, which form several groups. Their domain architectures depend on whether they have a conserved set of functional domains (composed of one or two subunits); thus, the family can be classified into classical (the calpains: 1, 2, 3, 8, 9, 11, 12, 13, and 14) or non-classical groups (missing domains DIII or DIV). Patterns of expression are used to classify the family into ubiquitous (the calpains: 1, 2, 5, 7, 10, 13, and 15) or tissue-specific types based on mRNA transcript levels in different tissues [1–3]. Two mammalian isoforms of the μ- and m-calpains (CAPN1 and CAPN2, respectively) are best characterized by the qualities that both of them are involved in both the classical and ubiquitous groups, and are each transcribed in most tissues. Another family member, calpain3 (CAPN3, p94) has specific expression in the mRNA of skeletal muscle, where its mRNA level is almost ten times greater than those of CAPN1 and CAPN2 [4]. The other tissue-specific CAPN members in humans are known as CAPN6 in the placenta and embryonic muscles, CAPN8 and CAPN9 in the gastro-intestinal tract, CAPN11 in the testis, and CAPN12 in the hair follicles [5].

CAPN subsists in the cytosol in inactive forms (non-Ca^2+^-binding) and dislodges from the membrane to the cytosol in response to an increased level of intracellular Ca^2+^. On the membrane, CAPN is activated in the presence of Ca^2+^ ions and phospholipids [6]. Activated CAPN decomposes its substrate proteins, in either the membrane or cytosol, by overcoming structural constraints imposed by catalytic domains released thereafter from the membranes. Several parameters that influence CAPN activity have been investigated to determine its roles in Ca^2+^-modulation, autoproteolysis, phosphorylation, and intracellular distribution and inhibition by its endogenous inhibitor calpastatin, (CAST). Only the CAPN2 regulation system was previously known to have both CAPN2/CAST-complex structure-function, and interaction sites [7–10]. Given the nature of sustained CAPN expression and intracellular Ca^2+^ levels, not all of the associations between CAPN activation and its pivotal, degenerative role in calpainopathies have been fully analyzed in relation to its biological properties, and there has been a lack of medical studies at the molecular level. Even in their primary roles, the CAPN family members belonging to different groups share homologous sequences (more than 50%) and domain structures in the protease domains DI and DII with a substrate binding cleft between them. These similarities, however, do not indicate similarity in physiological function or in biochemical properties. They differ in that they have unique distributions and different Ca^2+^-sensitivities [11,12] with corresponding binding residues, although many CAPN substrates are similar or overlap those of other members. Furthermore, the role of CAPN proteolysis is to modulate substrate structures and activity rather than simply cut them off. Importantly, this proteolysis is not explained by a consensus cleavage site, rather by recognition of a bond between domains and its particular conformational properties. Even so, the CAPN family shares similar catalytic-triad (cysteine, histidine, and asparagine residues) coordination in its activated form [13–15]. These forms and their nature have made it hard to predict the substrate proteins of CAPN proteolysis with any precision.

To precisely analyze the effects of the reduction or enhancement of CAPN activity, the inhibitory preferences of CAST conserved at the potential sites could be an important link between the candidate inhibitors. (CAST has four equivalent domains; each domain inhibits one CAPN molecule with variable efficiency). We thought that CAPN1 and CAPN2 might be regulated by CAST in different ways and that identical group members might also be regulated differently. In this study, we analyze comprehensive binding patterns and constraints among functional sites (binding contact regions with molecular partners and Ca^2+^-binding motifs, which play significant roles in the regulation of the CAPN response) using targeted virtual mutations to prove its specificities in the recognize companions. During the screening of inhibitor candidates, simulation of their inhibition mode effects should be carried out with the recognition that disease-related variation in the CAPN system brings out more structural than functional defects. Protein stability is crucial for physiological function, and many disease-associated cellular processes lead to protein destabilization and aggregation (amyloid diseases, limb-girdle muscular dystrophy caused by defects in CAPN3). These molecular characters influencing activity are directly related to the enzyme itself and the stability of the CAPN complex with CAST. Thus our research was adapted to the characteristics of the enzyme. Subsequently, our findings shed light on some aspects of improving the specificity and stability of potential peptide-analogous-inhibitors. We believe that by identifying the interaction preferences, the binding constraints on binding of the CAPN1 system with CAST, or activation factors, the results of this study will be of great value for informed decision-making in disease diagnosis using SNP markers in an early stage and using the specificity of the candidate endogenous and exogenous inhibitors of CAPN in the discovery phase.

## 2. Results

### 2.1. Structural Comparison between Active and Inactive Bovine CAPN1 Models

#### 2.1.1. Comparison of the Large and Small Subunits in CAPN1 and CAPN2 Molecules

In the present study, we predicted the molecular structures of bovine CAPN1 in the non-Ca^2+^-bound and Ca^2+^-bound forms as well as the inhibitory properties of the fourth inhibitory domain of bovine calpastatin (CAST 4 domain, CAST4; residues 583–710 in the bovine CAST) from the bovine CAPN1/CAST regulation system. It is well known that the CAPN1 and CAPN2 structures are heterodimers and multidomain enzymes, which contained large and small subunits for both CAPN molecules. The large subunits are made up of domains DI to DIV, and the small subunits contain two domains (DV as a Gly-Pro rich domain, and DVI). The large and small subunits of the CAPN molecule have been referred to as the catalytic and the regulatory subunit because the larger subunit itself could retain the proteolytic activity of the CAPN molecule and because the smaller subunit requires chaperone activity to facilitate proper folding of the larger subunit through interactions with the *N*-terminal anchor [16,17]. The small regulatory subunit of CAPN is identical in CAPN1 and CAPN2 molecules [18], and it seems to stabilize the non-covalent association of hydrophobic interactions in the *C*-terminal regions of DIV and DVI, which are partial domains of other CAPN molecules. It is well known that both CAPN subtypes also undergo Ca^2+^-induced autoproteolysis and that the autolysis sites are similar, but not identical, to those of other species. For bovine CAPN1, the *N*-terminal 15 amino acids are removed first to produce a 78 kDa intermediate followed by removal of a 12 amino acids to produce a 75 kDa from the large catalytic subunit (80 kDa). Also, the *N*-terminal 26 amino acids and the entire glycine-rich domain (more 28 amino acids) is removed from the small regulatory subunit to reduce the 28 kDa subunit to 18 kDa during autoproteolysis [19]. However, it is unclear whether autolysis has an important role in CAPN function or if it may only affect activated CAPN.

**Figure 1.**
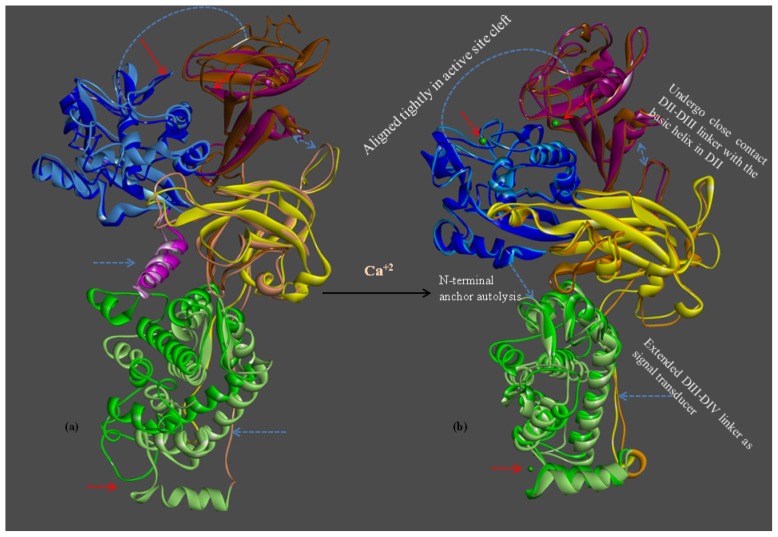
Bovine (CAPN1) and rat (CAPN2) structures in the absence of calcium (**a**) and bounded calcium (**b**); both large subunits with masses near 80 kDa subunits of CAPN1 and CAPN2 share 55%–65% sequence homology, and the structural similarities between them are very great. The protein structures all have the same domain color schemes: *N*-terminal (pink), DI (blue), DII (brown), DIII (yellow), DIV (green). The three Ca^2+^ ions are shown as green spheres. Superimposition of the modeled bovine CAPN1 (dark color) and rat CAPN2 (light color) structures reveals a high degree of structural homology in all forms. The majority of structural deviations are superimposed, large catalytic subunits with and without Ca^2+^-binding. The conformational changes of both structures have been shown to resemble other CAPN isomers.

Upon homology modeling, the bovine CAPN1 model structure was built in only large catalytic subunits. The modeled catalytic subunit of bovine CAPN1 consists of multi-domains of approximately 700 amino acid residues having four domains (from DI to DIV, DI: residues 30–220; DII: 221–386; DIII: 387–543; DIV 544–716). There is also an EF-hands structure characteristic (each of which Ca^2+^-binding helix-loop-helix structural motif is characterized by a common building block, EF-hand 1; residues 543–578, EF-hand 2; 587–620, EF-hand 3; 617–652, EF-hand 4; 682–716), of *N*-terminal α-helix (from Tyr10 to Gly29) in inactive (Figure 1a), but not active forms (Figure 1b). Although it is not apparent from the modeled 3D structure (Figure 1), DIV is composed of five EF-hand motifs, of which the fifth EF-hand motif contributes to the major heterodimer interface (putative corresponding residues on the bovine CAPN1 large subunit; Leu680, Met686, Phe687, Phe690, Trp708, Leu709, Leu711, Met713, Phe714) within each of the penta-EF-hand domains (DIV and DVI of the catalytic large and regulatory small subunits, respectively). In both CAPN2 crystal structures [18,20,21], although DIV also has the same secondary and tertiary structures as the small subunit DVI before or after the binding of Ca^2+^ ions, this permits valuable information to be gained about both DIV itself and, effective interaction interfaces in both the domains or between CAST4 subdomain C and the CAPN1 DIV with such high structural similarity between them. It would be reasonable to regard similar binding modes and Ca^2+^-induced conformational effects for CAPN interdimer contacts as seen in the CAPN2’s DIV/DVI heterodimer structures [22–24].

The bovine CAPN1 has been very well preserved at 89.5%–94.8% protein sequence identity and 95.3%–98.2% similarity in the overall catalytic subunit (four domains DI to DIV and *N*-terminal anchor helix region). This is comparable to those of other mammalian CAPN1s. In particular, the bovine CAPN1 had 94.7% sequence identity and 98.2% similarity with human CAPN1, corresponding to 89.7% sequence identity and 95.3% similarity for rat CAPN1, of which the substructures of the larger catalytic subunits were previously known. As seen in Table 1, the larger catalytic subunit of bovine CAPN1 is significantly homologous to that of the mammalian CAPN1. In terms of domain conservation, DIV was most highly conserved, having 93.6%–98.3% sequence identity to the DIV of mammalian CAPN1; DI and DIII from bovine CAPN1 had 91.6%–95.3% and 87.3%–94.3% sequence identity, respectively, with their counterparts from mammalian CAPN1. The range for DII was 84.9%–93.4%; thus, it was also well conserved. The α-helical *N*-terminal anchor sequence of bovine CAPN1 is short (19 amino acids) and its makes contact only with DIV but not with any other domains in our structural model; moreover, the *N*-terminal anchor among the different species shows up to 86% sequence conservation. Moreover, the catalytic subunits of bovine CAPN1 and CAPN2 are different gene products (genes on chromosomes 29 and 16, respectively), but they share 62.5% sequence identity and 81.5% similarity, particularly a 62.4% sequence identity to humans (corresponding to sharing 55%–65% sequence homology of both CAPN subgroup catalytic subunits within a given other mammalian species [10,25]). It is very interesting that DIV shows the highest similarity among catalytic subunits when compared to the same CAPN subgroup (between mammalian CAPN1), but the protease cores are very similar (77.0% for DI and 65.7% for DII) when different bovine CAPN subgroups (CAPN1 and CAPN2) are compared (Table 1). In addition, the catalytic domains of rat and human CAPN1 and CAPN2 show a high degree of identity (93%) between rat and human CAPN1 of DIV. On the other hand, the CAPN1 protease cores (DI and DII) of these two species did show a high sequence identity (87% in DI and 70% in DII) of rat CAPN1 and human CAPN2. This tendency is maintained to DIV, and the protease cores are the most conserved regions within the same-animal CAPN group and within a given CAPN subgroup (CAPN1 or CAPN2) among different mammalian species, too. Furthermore, the active site clefts within a protease core are very well conserved throughout the mammalian CAPN group. Therefore, it is reasonable to suggest that both CAPN subgroups have very similar, if not identical, substrate-subsite specificity for proteolysis function [26–29].

#### 2.1.2. Evaluation of Homology Modeled Proteins

**Figure 2.**
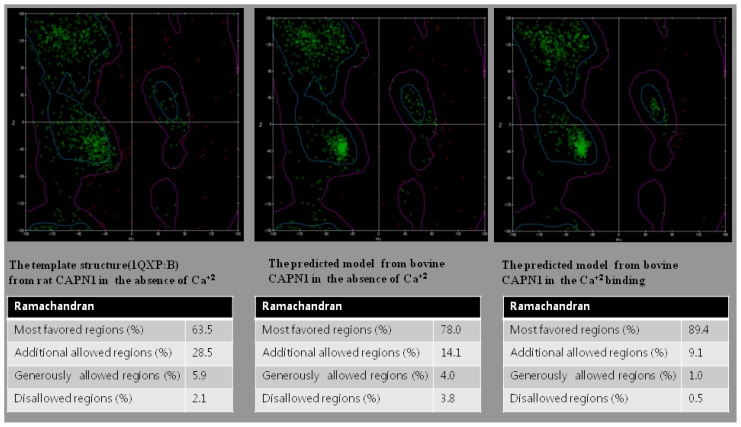
A structural analysis of the template structure (1QXP:B) from rat and bovine, (inactive and active) CAPN1 structural models by Ramachandran plot, a graphical representation of the local backbone conformation for phi and psi torsion angles of each residue in a protein. Amino acid types are represented graphically: glycine as triangles, proline as squares, and all other types as circles. In the side region (blue contour), acceptable regions are at least within the boundary of the overlap 10% regions on the other hand outside region (at pink contour) represent residues outside the acceptable regions outside the boundary of the overlap 10% region.

In homology modeling of both bovine CAPN1 and its regulation system, the predicted model structures are selected by the total energy of the PDFs. If the generated models all have similar PDF total energies, we use the DOPE score based on statistical potential. The model having the lowest PDF total energy and DOPE score was chosen as the final model that was optimized in the relationships of both structural features of template protein structures. In addition, the final model was subjected to a series test for its validation using structural assessment by the Swiss-model. The model validation was carried out for both local and global model-quality evaluation, stereochemistry, and structural features analysis to further measure its suitability. Figure 2 and Table 2 show the assessment results of the modeled structures for active and inactive bovine CAPN1 from its protein sequence along with the structural analysis by Ramachandran plots [30]. In Figure 2, the plot includes a representation of the favorable and unfavorable regions for all residues, used as a first check to verify predicted torsion angles in the protein models and thus determine if individual residues are likely to be built correctly. The integrity of the model molecule is highly dependent on the quality of the template protein structures (B chains of PDB code 1QXP and 1KXR, 1QXP:B and 1KXR:B) and whether or not individual residues are likely to meet crystallographic benchmarks (e.g., bond length, valence angles). The results of the residue-backbone conformations of our models were, thus, compared with the template structures by monitoring the deviation from known values gathered from high-resolution crystal reference structures (Ramachandran plots in Figure 2 and two agreement terms of the QMEAN6 score in Table 2). Figure 2 clearly shows that the stereo-chemical qualities of the predicted CAPN1 models were built on 78.0% and 89.4% in the most favored regions of the plot for non- Ca^2+^-bound (inactive) and Ca^2+^-bound (active) forms, respectively. Furthermore, the predicted secondary structure and solvent accessibility in the two agreement terms of the QMEAN6 scoring-function supported higher values (80.2%, 74.9% for inactive CAPN1, 80.5%, 78.2% for active CAPN1 from bovine) rather than the two template structures (except 89.4% solvent accessibility agreement term of 1KXR:B), meaning good quality models as seen in Table 2.

**Figure 3.**
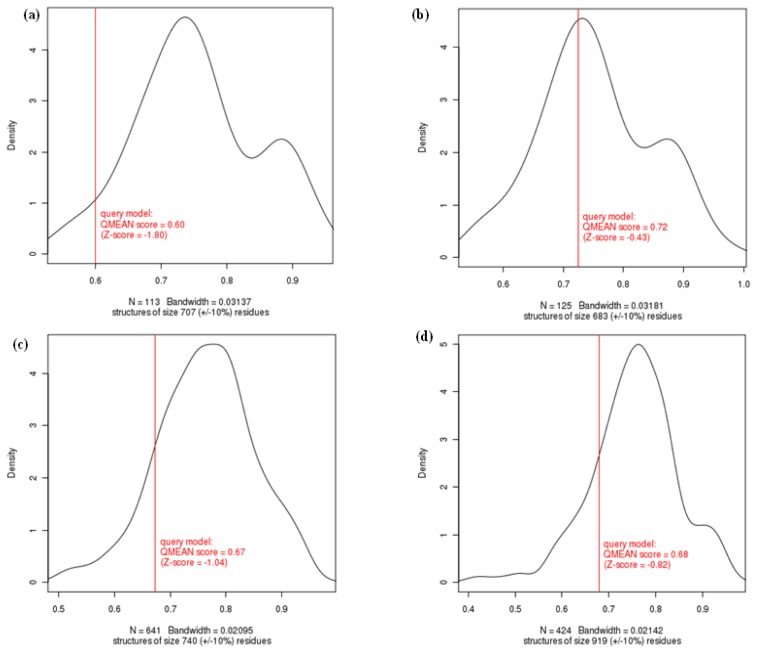
Density plot of predicted models and the template structure (3BOW) provide a measure of quality by relating models to reference structures of similar size solved by the result of X-ray crystallography that reveal how many standard deviations the model QMEAN score differs from the expected values for experimental structures. These are (**a**) inactive bovine CAPN1; (**b**) Ca^2+^-induced active bovine CAPN1; (**c**) regulation system of a catalytic subunit CAPN1 and subdomains AB CAST4 complex from bovines; and (**d**) the CAPN2/CAST4 complex from rat as 3BOW.

Other model quality tests were indirectly compared using the pseudo energies of the contributing terms of QMEAN6 [31]. Since the QMEAN6 score is protein-size dependent and larger proteins tend to have higher scores, our models seem likely to have medium qualities compared with the template structures in Table 2. There, the QMEAN *Z*-score is a score normalized to a mean of 0 as the average quality of high-resolution X-ray structures and a standard deviation of 1, identifying geometrical features like PDF total energy. Unlike QMEAN6, the QMEAN *Z*-core directly shows that the model QMEAN score differs by −1.80 and −0.43 standard deviations from the expected values for the 113 reference structures with 707 ± 10 residues in the inactive CAPN1 form, and 125 reference structures with 683 ± 10 residues in the active CAPN1 form Figure 3a,b. The density plot in Figure 3 shows the middle based on the QMEAN scores of a given model with respect to the reference sets. In this density plot, the reference structures are a non-redundant subset of the PDB, sharing less than 30% pair-sequence-identity with the same protein-size against target sequence, and they are solved at a resolution of less than 2 Å. Each list of reference structures is not shown but the number of reference models used in the calculation is shown at the bottom of the plot in Figure 3. As the QMEAN scores of the model structures had a very close approximation to the average scores of each non-redundant set of high-resolution X-rays structures of similar size, both forms of our predictive models could be regarded as good structures.

#### 2.1.3. Overlay Structure Similarity Using Field-Based Alignment

Furthermore, to compare and analyze the structural features of the bovine CAPN1, the predicted models were superimposed over the rat CAPN1 template structures (1QXP:B, 1KXR:B) using a combination structural overlay of both by field alignment based on maximizing steric and electrostatic repulsion between atoms [32], and by target alignment to bovine CAPN1 based on all atoms. The bovine CAPN1 model structure, compared to the template structures, has a high overlay similarity (0.567) with 1QXP:B of overall structure for the inactive form, *vs.* 0.411 in the only protease core (DI and DII) with 1KXR:B for an active form, based on all atoms (not shown). This too demonstrates the reliability of the predictive models too. On the other hand, the inactive bovine CAPN1 (Figure 1a) was different in the alignment of the local active sites and global structural architecture on the active structure (Figure 1b) where the structures being aligned were allowed to superimpose as 0.407 overlay similarity for all atoms in the full-length form. This could be closely related to the Ca^2+^-induced conformational changes leading the bovine CAPN1 to produce active conformers not only of the enzyme itself, but also of its available substrates or enzyme inhibitors, such as CAST.

#### 2.1.4. Potential Functional Residues and Interaction Interfaces

**Figure 4.**
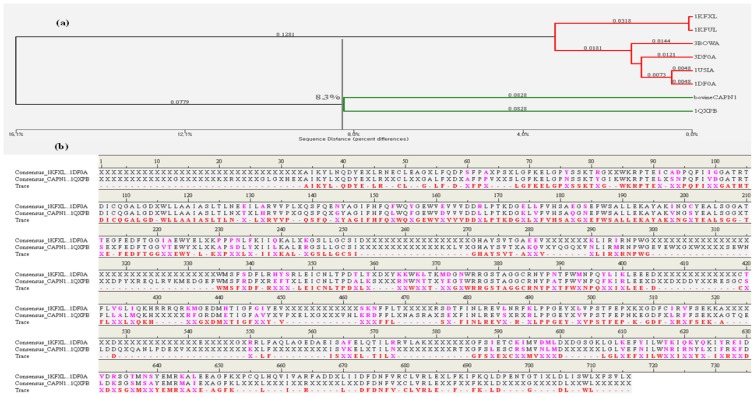
The residue conservation pattern in protein subgroups (CAPN1 and CAPN2) with protein structure: (**a**) A view of similarities in aligned dendrogram sequences with dendrogram, in which the CAPN can be divided into subgroups (CAPN1-green and CAPN2-red) at an 8.3% distance cutoff and (**b**) A segment of CAPN sequence for two subgroups (CAPN1 and CAPN2) for which the conserved residues (red) and class-specific residues (pink) are defined as trace residues from a multiple sequence alignment and a structure alignment based on the cutoff value. To avoid numbering trace residues, all positions are described in terms of the bovine CAPN1 amino acid sequence.

It is essential to identify protein functional residues and interaction interfaces (e.g., the proteolytic core or molecular counter-partner binding sites) in order to understand how the bovine CAPN1 carries out its proteolytic function, and how the bovine CAST4 domain recognizes Ca^2+^-binding and then induces the active CAPN1 structure into forming the CAPN1/CAST4 complex. In our research system, functional interfaces can also serve as targets to study residues, which are likely to be important to the bovine CAPN protease function and functional specificity of a given CAPN1 subgroup. A direct connection between conserved residues in a CAPN group and their functional importance was made from both by multiple sequence alignment then by mapping the alignment sequences to known 3D-structures among mammals using the hierarchical clustering method [33] based on pairwise residue distance between specific side-chain atoms. This also defined the functional specificity of the subgroups by partitioning the CAPN protein sequence alignment into each subgroup as CAPN1 and CAPN2 at a selected sequence-distance cutoff according to their sequence homologues. CAPN1, with different subgroups from CAPN2, may have similar functional residues as conserved residues but different functional specificity as class-specific residues. As seen in Figure 4a, at a given sequence identity of 8.3% between CAPN1 and CAPN2, each subgroup consists of smaller members and shows more functional specificity (Figure 4b).

**Figure 5.**
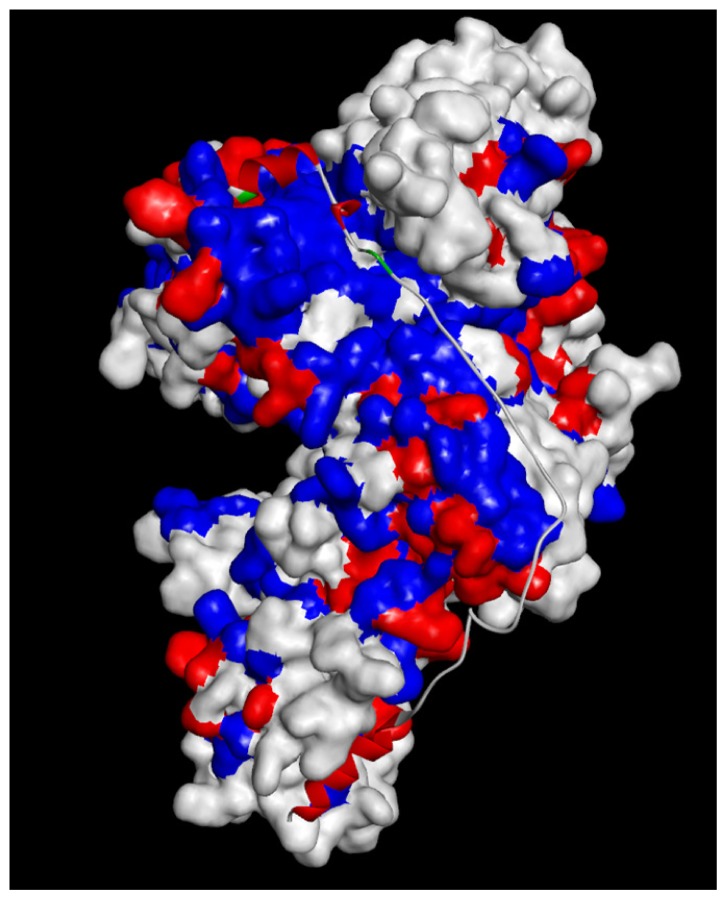
Analysis of residue conservation pattern in a CAPN group with protein structure to identify interaction interface (e.g., binding site of subdomain AB of CAST4). Trace residues may be conserved residues (blue), and class-specific residues for a CAPN group (red), or non-trace residues (white). For the Ca^2+^-dependent bovine CAPN1/CAST4 regulation system, the binding pockets of DI-DII and DIV are conserved sites, each of which subdomain A and B of CAST4 domain attracts site, in contrast to the DIII interaction surface provide class-specific and inter-variable specific sites among CAPN1 subgroup to the part of its subdomain B.

The residue conservation patterns of bovine CAPN1 represented by trace residues in Figure 4b are considered to be important for Ca^2+^-binding sites, cysteine protease functions, or CAPN1 folding into different active and inactivate structural architectures. Since protein structure is more conserved than the sequence, especially at the interaction interface, those trace residues can be further analyzed by mapping the conservation patterns to the CAPN1 model structure, by clustering exposed or buried trace residues in its aqueous environment (Figure 5). In this respect, the effects of bovine CAPN1 trace residues are designed to extract functional residues to be responsible for binding to its molecular partner or for enzyme activity in CAPN1 itself and in the CAPN1/CAST proteolysis regulation system, and to distinguish the specificity of the CAPN1 functional residue from that of CAPN2.

#### 2.1.5. Comparison of Ca^2+^-Binding Patterns in CAPN Subgroups

**Figure 6.**
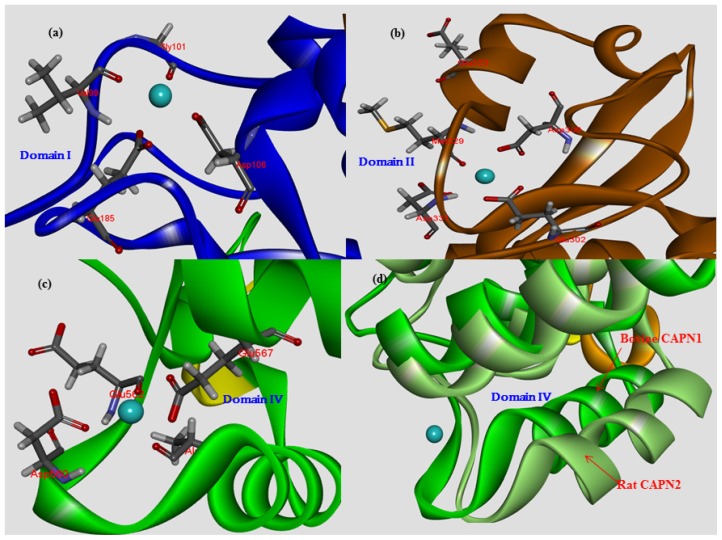
Three conserved Ca^2+^ binding sites in the bovine CAPN1: (**a**) the loop in DI provides Ca^2+^-chelating residue side-chains of Asp106 and Glu185 and the backbone oxygens of Val99 and Gly101; (**b**) the second loop of the Ca^2+^-binding site in DII made by the side chain of Asp309, Glu302, the backbone oxygens of Glu333 and Met329, and the backbone nitrogen of Asp331; (**c**) The loop region of EF-hands has several acidic residues (Glu562, Asp560, and Glu567) and the backbone oxygen of Ala557 that help coordinate the Ca^2+^ ion; (**d**) Ca^2+^ binding to EF-hands in DIV, not only an equivalent location but also conserved binding pattern ADEE for both sub-groups (CAPN1 and CAPN2).

Bovine CAPN1 has multiple calcium binding sites. We preferentially applied the trace residues to an analysis of Ca^2+^-binding sites in the CAPN subgroups (between CAPN1 and CAPN2). The active bovine CAPN1 model structure bonded to three Ca^2+^ ions at the three conserved sites. Among these, two Ca^2+^ ions bound in the protease core (DI and DII) were at the class-specific residues of the conservation pattern in each subgroup (either CAPN1 or CAPN2) in particular to one Ca^2+^-binding to the EF-hand containing DIV [6] is at conserved residues across the mammalian CAPN group (Figure 4b and Table 3). Table 3 shows conserved patterns of Ca^2+^-binding for subgroups represented by the trace residues and the partial sequences of the CAPN catalytic subunit; clustered into different Ca^2+^-binding sites. The first active site of the Ca^2+^-binding pattern is XGED when X is the valine/isoleucine variation. This represents a relatively conservative substitution of a hydrophobic amino acid in either the CAPN1 or CAPN2 subgroup at the X position in DI (Figure 6a). In contrast, the second EDXDE binding pattern of Ca^2+^ ions (active site 2 in Table 3, Figure 6b) in DII is conserved residues in the CAPN1 subgroup. There, all member sequences have methionine at the X position, whereas the residue at this X position in the CAPN2 subgroup (Figure 4b), responsible for functional selectivity, is forced to mutate in the presence of histidine into glutamine, as a relatively diversiform polar amino acid from acidic to basic properties among mammals during its evolution to make intra-distinction. This X residue variation located in DII of the CAPN2 subgroup, could alter the Ca^2+^-binding affinity required to initiate protease activity for both members of the CAPN2 subgroup, which then might affect the enzyme functional selectivity. Both Ca^2+^-binding patterns (Figure 1, red dotted arrows) appear to occupy surface-accessible positions derived from two peptide loops (residues 96–108 loop and residues 328–332 loop). Though defined as a significant structural difference between the active and inactive forms of the protease core region within the CAPN group, the protease core is nearly identical to both the peptide loop structures within the active CAPN1 subgroups in the presence of Ca^2+^ ions (Figure 1b). These two Ca^2+^ ions, which bond in the enzyme proteolytic core, must facilitate the rearrangement of a catalytic triad located at the interface between the protease domains DI and DII, which are more substrate binding pocket into an active conformation [34]. As shown in Figure 6c,d, the third Ca^2+^-binding pattern (ADEE) is not only highly conserved among the species but also structurally characteristic in the CAPN group. This pattern is also found at the segment of the Ca^2+^-binding EF-hands motif (EF-hand 1; residues 543–578 for bovine CAPN1), and it appears to be a fixed feature as negatively charged amino acids at a pH level of 7.4 for characterization of a Ca^2+^-binding site. It is conceivable that the conserved Ca^2+^-binding patterns of bovine CAPN1 can provide very important insights into the binding patterns, which may not be attributed to large, local conformational changes of the Ca^2+^-binding sites in both the CAPN1 and CAPN2 subgroups but may rather have functional specificity of interaction at the interface with Ca^2+^ ion on the basis of molecular structure. This is well represented in the structural superimposition (Figure 1b) in which the active structures between rat CAPN2 (template structure 3BOW:A) and the model derived from the bovine CAPN1 model overlapped such that the active Ca^2+^-binding sites were at highly con*s*erved positions on the structure alignment of Ca^2+^-binding pattern residues within 3.5 Å from one Ca^2+^ ion (Table 3).

#### 2.1.6. The Role of Structural Water Molecules for Their Ca^2+^-Binding Patterns

It could be valuable to analyze the Ca^2+^-binding patterns to determine whether water molecules are essential for divalent ions to be effectively bonded to the CAPN1 enzyme or not. Thus, there is considerable interest in how specific water molecules might be involved in chelation of one Ca^2+^ ion by neighboring Ca^2+^-binding residues. For example, two ordered water molecules in known active CAPN structures are held by restraining interactions, from the neighboring Ca^2+^-chelating side-chains of Glu185 and Gly101. Such residues are conserved in all CAPN groups at the positions corresponding to Glu175 and Gly91 in rat CAPN2 (Table 3), and within the Ca^2+^-binding pattern as XGED in DI. Strangely, the two water molecules [35] (HOH450, HOH451 in the 1KXR structure) offered the Ca^2+^-binding equatorial coordination in the active rat CAPN1 structure (1KXR). In contrast, another water molecule (HOH380) included in the Ca^2+^-binding pattern of EDXDE in DII, exhibits pentagonal bipyramid coordination for the same 1KXR template structure. In other cases, two water molecules are components of eight coordination to Ca^2+^-bound DI, and another one provides seventh coordination in DII for the 1KXR structure. Most EF-hands motifs provide seven coordinating a pentagonal bipyramid configuration to Ca^2+^-binding as it is in the activate CAPN2 structure from rat (as both Ca^2+^ 401 and 402-binding coordination residues in 3DF0 structure) and would be the third Ca^2+^-binding pattern ADEE in DIV of the current study.

Thus, many X-ray activated CAPN1 structures are an excellent starting point for modeling studies to assess whether the modeling reflected how changes in Ca^2+^-coordination sites can affect the Ca^2+^-binding geometry, depending on the relative locations of the water molecules. In addition, it is generally considered an explicit hydration effect that water molecules that form Ca^2+^-coordination sites are positioned at exact locations in the modeling process. However, in our structural models, water molecules are considered in the empirical scoring function as dielectric constant 80, for solvation effects that address this problem. This is due to the lack of currently available known structures of active CAPN1that could be used to extract common features for Ca^2+^-binding geometry in relation to the locations of water molecule in mammalian CAPN. It is still unclear whether these water molecules directly participate in Ca^2+^-binding within other CAPN1 species. In this study, our model did not represent the geometry of each Ca^2+^-binding pattern or, the *H*-bonding stabilization effects induced by Ca^2+^-coordinating residues with other water molecules to form the Ca^2+^ ions/CAPN1 coordinated complex. In another aspect, neither the Ca^2+^-bound structure model represented by a lower Ca^2+^-requirement than CAPN2, nor a much more Ca^2+^-binding candidate’s patterns particular to an EF-hands motif (EF-hand 2–4) are more extract from clustering functional residues across the mammalian CAPN group. This is apparent due to the Ca^2+^-binding pattern differences between subgroups. Some potential limitations of this approach arise from the very small number of known, full-length, active CAPN structures that might be used to answer the question.

Fortunately, the Ca^2+^-binding pattern information was taken together with both the predicted changes in the active CAPN1 itself or the stability of CAST/CAPN1 complex, and the binding affinities of the CAST inhibitor and the Ca^2+^ ion, by evaluating mutation effects *in silico*. In addition, we reasoned that the CAPN1 subgroup has different Ca^2+^-binding characteristics from those of CAPN2, which make some contribution to its activation. This is because the variety of configurations of the Ca^2+^-binding sites of CAPN group is rather limited on the most common site as the EF-hands motif if it is not positioned on domain DI, DII, and DIII. In our approach, two Ca^2+^-binding patterns to other EF-hands in some of DIV distinct from the CAPN2 subgroup may be DDXKE (active site 4 in Table 3) and DDSXE (active site 5 in Table 3) of the CAPN1, while the DN pattern (active site 6 in Table 3) will be highly conserved across the mammalian CAPN group. For the DDXKE pattern of Ca^2+^-binding to EF-hands in DIV, the class-specific residue at the X position existed as the conservative polar amino acid asparagine for the CAPN1 subgroup, and as serine for the CAPN2 subgroup. In addition, the other DDSXE pattern was preserved in the subgroups so that the X position residue has an alcohol functional group side chain (serine and threonine for CAPN1 and CAPN2, respectively). Thus, it is likely that the three observed Ca^2+^-binding patterns (active sites 3–5 in Table 3) at the EF-hands motif in DIV are maintained as polar and acidic properties across the mammalian CAPN group, and that their Ca^2+^-dependent protease activities are greatly affected by other exposed interactional residues for Ca^2+^-binding from the active conformation. Since the Ca^2+^-induced conformational changes in the EF-hands would be stabilized by the H-bond networks and extensive hydrophobic contacts, two Ca^2+^-bound EF-hands are more immobilized than a single Ca^2+^-bound EF-hand without any long-range energetically unfavorable helical movements [36,37].

In this analysis of the Ca^2+^-binding pattern, it is unclear how many Ca^2+^ ions are bound on the bovine CAPN1, raising questions as to whether Ca^2+^-binding at some site in addition to, or other than, the EF-hands affects the proteolytic activity of CAPN1, but the former three of these six Ca^2+^-binding properties on the conserved binding patterns are well characterized by significant amino acids at both the class-specific subgroup positions and at the conserved binding positions of the mammalian CAPN group. A further interesting point is that given high-resolution X-ray and NMR investigation of these structures, an accurate description of the Ca^2+^-binding sites should permit further theoretical work on the coordination of calcium caused by stereochemical constraints. These might then inform models for the structure-functional relationships of intracellular Ca^2+^ receptors, which enable them to reversibly bind to their target proteins and regulate their proteolysis activity.

#### 2.1.7. Structure Flexibility and Changes on CAPN Activation

Many previous CAPN structural and biochemical studies have supported the prevailing approach in which Ca^2+^-induced, CAPN activation effects are represented in terms of enzyme structural features: (i) intra-domain conformational changes of the protease core result in closer arrangement to a catalytic triad (Cys, His, Asn) forming an active protease; (ii) an *N*-terminal anchor hangs down upon Ca^2+^-bound CAPN activation thereby reducing hydrophobic contacts with DVI caused by the lack of the restraining interaction from neighboring domain DI through the anchor peptide thus allowing DVI to be more flexible; (iii) the linking residues connecting DIII to DIV are extended and act as signal transducers; (iv) DIII mediates induced conformational changes from Ca^2+^-binding to EF-hands in DIV to DII, which results in stronger electrostatic interactions at the interaction interface between the loop of several acidic residues in DIII and the helix of basic residues in DII, which then forms a closer contact DII-DIII link to the basic helix in DII; (v) though the Ca^2+^-binding to EF-hands in DIV induces smaller intra-conformational changes with more flexibility while reducing compact to each other EF-hands than non-Ca^2+^-bound form. In our model structures, several important domain motions as the most significant differences between the bovine CAPN1’s active and inactive states were well predicted in cooperative conformational changes at the multiple sites mentioned above. Moreover, the idea that a complementary movement occurred via signal transmitted from DIV through the link to DIII was satisfactorily demonstrated. This is shown for the Ca^2+^-induced structural changes during CAPN activation (Figure 1).

**Figure 7.**
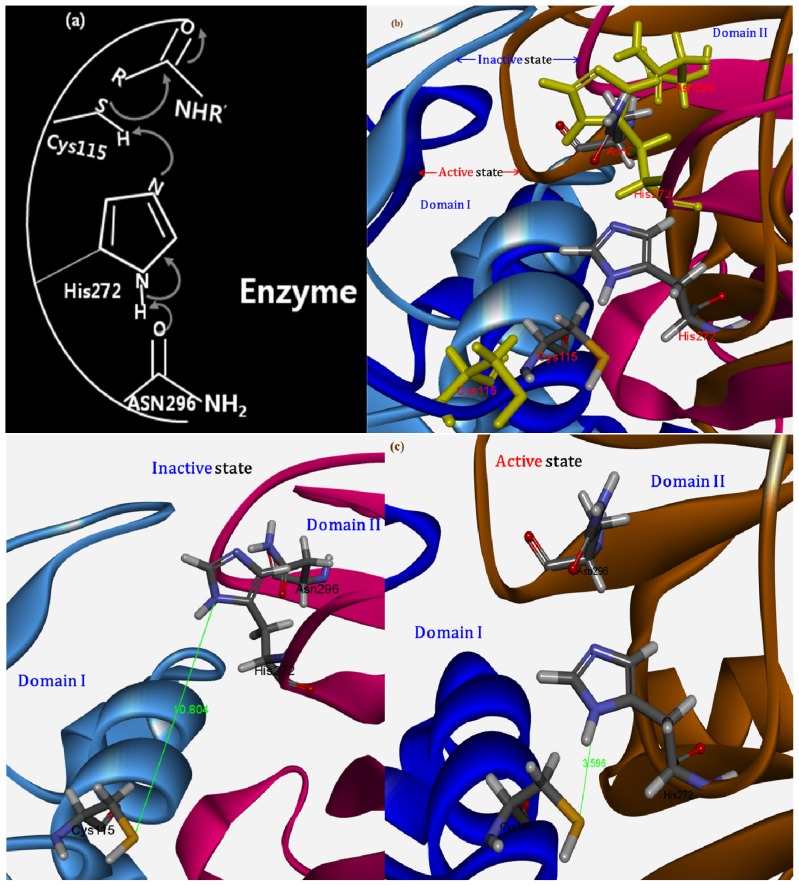
Ca^2+^-binding induced conformational changes in active site region of bovine CAPN1. (**a**) The mechanism of a catalytic triad facilitates the hydrolysis of substrate amide bond; the critical step of the catalytic process involves formation of a reactive thiolate/imidazolium ion pair for histidine to deprotonate the cysteine and activate it for nucleophilic attack on a substrate (adapted from Abell [38]); (**b**) Stereo view of residues (Cys115, His272, and ASN296) between non-Ca^2+^-binding (yellow) and Ca^2+^-binding bovine CAPN1 (colored by element); (**c**) Cys115, His272, and Asn296 are highlighted in a ball- and-stick representation. The functional active site cleft between the core domains is radically repositioning before and after activation. Ca^2+^-binding induced a large and significant conformational change of active site that brought positions of the catalytic residues to within 3.6 Å of each other (10.8 Å separates them in the absence of Ca^2+^ structures of the bovine CAPN1 molecule.

Cysteine proteases typically have an active site cleft in a common catalytic cysteine and histidine, and the bovine CAPN itself also has a catalytic triad as a characteristic cysteine protease. The active site of this enzyme contains a Cys115 in DI, a His272, and an Asn296 in DII. These catalytic residues are located at the interface between DI and DII. The Ca^2+^-induced activation results in the significant arrangement of a catalytic triad as a conformation with a shorter distance between the His272 and Cys115 residues, which facilitates the hydrolysis of amide bonds in peptide substrates (Figure 7a,b). In the homology modeling process, we approximately considered the catalytic ion-pair of cysteine and histidine residues in active sites for the deprotonation of Cys115 and protonation of His272 by filling valences of hetero-atoms and adding polar hydrogen in the optimization of the local activate conformer. This enables an attack on the sulphur of the nucleophilic cysteine by the formation of active thiol sites (key residues are illustrated in Figure 7a). Within the predicted structures of the model, Ca^2+^-binding induced conformational changes that brought the distance of the catalytic Cys115 and His272 residues to within 3.6 Å (Figure 7c) of each other. This is compared to a distance of 10.8 Å for the same residues in the unbounded Ca^2+^ model structure (Figure 7c) of the CAPN1 molecule. When activated by Ca^2+^ ions, the parts of the protease core between DI and DII are moved closer to each other, but the relevant secondary structure does not change. However, this leads to considerable steric hindrance in the catalytic cleft, mainly caused by the Pro297 and Trp298 loop, in addition to the active site residues (Cys115, His272, Asn296 and Trp116). Furthermore, the acidic residues of Asp106 and Gln182 in DI would then come in close contact with Glu330 and Asp331 in DII, so that the charge repulsion caused is somewhat less than that for the CAPN2 subgroup (the residue mutation from Glu172 in CAPN2 to Gln182 in CAPN1) at the same positions in active bovine CAPN1. Specifically, in our model, promotion of productive folding of the large catalytic subunit was well reflected in the assembled geometry and in the induced effects of the catalytic triad during the activation of the bovine CAPN.

**Figure 8.**
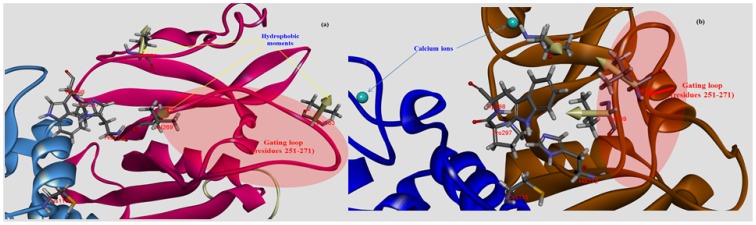
The key movements of Cys115 close to His272 for deprotonation of the former to occur involve a rotation of Trp298 in DII (**a**) Trp298 and Pro297 residues of the bovine CAPN1 in the inactive conformation blocking the active site and the adjacent equivalent region of Trp298 and Pro297 residues; (**b**) At the same time as undergoing major conformational changes upon activation, the Trp298 residue shifts to form an exposed position in the cleft to intrude into a hydrophobic patch formed by the rearrangement of the Ca^2+^-binding loops. Thus, the side chains of hydrophobic residues (Val269, Val301, and Val 263) reorient towards the interior Trp298 to give some indication of the preferred orientation of the hydrophobic moment vectors to facilitate stabilizing Trp298 in the bovine CAPN1 protease core due to cooperativity of these residues and open conformer of the flexible loop (residues 251–271) in DII from the protease core cleft.

In the Ca^2+^-binding to activate the protease core, the short anti-parallel sheets (β8–β9, residues 160–179) in DI and the flexible peptide loop rearrangement (residues 96–108) between the two catalytic domains (DI and DII) allow the exposed hydrophobic pockets to be nearly stabilized by hydrophobic interactions, where the aromatic side chain of Trp298 residue in DII of displacement increases hydrophobic moments through hydrophobic contacts with both aliphatic side chains of two conserved Val269 and Val301, and closely adjacent Val263 as seen Figure 8. Moreover, the π-stacking interaction of both side chains in Trp298 and His272 is not parallel; rather, it contributes to the stabilization of the hydrophobic pocket for proteolytic activity of the bovine CAPN1 enzyme (Figure 8b). For the π-stacking interaction of both the Trp298 and His272 residues, the distance between the centroid of each pair of π-rings is within 6 Å, and the angle between the normal of both rings and the centroid-centroid vector falls to 45°. Therefore, the π-stacking is done by Trp298 in the enzyme-active-conformer. This helps to protect His272 and Asn296 from water-exposure and enables intra-assembly by the active site, which then facilitates catalysis. In the absence of Ca^2+^, the Trp298 and Pro297 residues enter the interaction interface of the protease core (between DI and DII). Because these residues reorient such that they lie across the core, they interfere with the assembly of the active site. The residues thereby prevent the non-Ca^2+^-bound, bovine CAPN1 from attaining an active conformer due to major steric hindrances as well as other cysteine protease structures (Figure 8a). Due to the hydrophobic interactions of three hydrophobic residues (Val269, Val301, and Val263) located in close proximity to the indole ring of the Trp298 residue, the Ca^2+^-bound structure facilitates an open conformation of the flexible loop of DII (residues 251–271) from the protease core, which is considered the gate to the catalytic cleft. In addition, the warped loop-conformer in an activated model structure (Figure 8b) serves to effectively reorient the hydrophobic residues towards Trp298 by arraying the hydrophobic moment vectors in one direction and by increasing the total sum of the moment vectors, while those for an annular loop conformer point in each staggered orientation in the non-Ca^2+^-bound structure (Figure 8a) into being ineffective.

The flexible gate loop (residues 251–271 in DII of bovine CAPN1) shows the highest dissimilarity of sequence and the most variable length among the CAPN subgroups (Figure 4b). In other words, the gate loop does not have conserved residues or class-specific trace residues among the CAPN group. In contrast, the loop (residues 96–108) in DI observed at the active site; made up of highly conserved residues (the QFIXXGATRTDIC amino acid sequence) across the CAPN group is color-coded red among the trace residues (Figure 4b). The former loop is quite flexible when present on the solvent-exposed surface, and it reveals major differences of the CAPN1 protease core and its inhibitor-complex structures (PDB code 1TL9, 1TLO) compared to their native active structures (non-bound inhibitor structures). The letter loop shows minor conformational change so that, between them, they make up a rigid surface that serves as the foundation of the active site [39]. This is well supported by the conserved patterns of our trace residue at their position across the CAPN subgroups (Figures 4b and 5). More interestingly, the former gate loop that is in contact with the inhibitor CAST or CAPN1 substrate may be a good target candidate for screening site-direct inhibitors focused on the selectivity and specificity of the bovine CAPN1. This might be done by defining the spacing cavity made from critical, active site residues. Since the hydrophobic pocket formed by Ala262, Val263, and Val269 observed only in bovine CAPN1 is not as shallow as that of the other mammalian samples in which Val263 is changed to Ile263, the hydrophobic interaction interface could come into contact with the unique structure of the inhibitor binding pocket following Ca^2+^-dependent activation.

As another important feature, the flexibility of the glycine rich region (Gly207–Gly220 residues) permits the side chain of Trp116 in DII to swing around and into the active site pocket. It was shown to be stabilized by the interaction between the glycine rich region and the conserved Arg residues (431, 434, 483, and 514, respectively) of domain DIII; it is not susceptible to interference from the Trp116 residue, unlike its non-Ca^2+^-binding counterparts. Interestingly, the interaction interface has no absolute specificity for the enzyme class-specific, which is highly conserved across the CAPN1 and CAPN2 subgroups. However, it has been regarded as a very important region corresponding to the inactivating effect of disease-causing p94 (The skeletal-muscle-specific member of the CAPN family: CAPN3, the CAPN isoform, can be regarded as close to CAPN1 and CAPN2, at least with respect to 45% sequence identity) gene mutations in LGMD-2A (limb girdle muscular dystrophy type 2A) patients [9,40,41]. More investigation is needed to know whether the gene mutation occurs in bovines. Structurally, the stability of this region, which contacts the narrow cleft within the protease core between CAPN1 and CAST4 subdomain B, plays a critical role as an indicator of the detailed CAST specificity for the enzyme. These are different interaction sites with the enzyme inhibitor among those of mammalian CAPN, which are directly related to various Ca^2+^-binding sites that play a part in regulating the biological functions of the enzyme and are sufficient to specifically target an inhibitor by considering both the depth and width of the bovine CAPN1 active -site cleft.

In the presence of Ca^2+^ ions, the EF-hands motifs in DIV-DVI have increased flexibility with the extending linker (residues 529–546) of DIII to DIV, and they lead to the release of the *N*-terminal anchor from DVI (domain DIV in our model structure. It was possible to the anchor helix adjacent to the hydrophobic surface of DIV in either direction.) whereupon the anchor helix is cleaved during its autolysis. This also triggers conformational changes that gradually weaken the interactions between the *N*-terminal anchor helix. DVI (DIV in our model structure) would directly affect the neighboring DIII through the linker (Figure 1), acting as an intramolecular signal transducer. Domain DIII of bovine CAPN1 contains two interaction interfaces within neighboring domains (DII and DIV). Each interaction interface is positioned at the DII/DIII (residues 328–348) and the DIII/DIV (residues 556–567) adjacent interfaces, directly linking the Ca^2+^-binding domain DIV to the catalytic domain DII. Therewith, DIII could be signaled by both catalytic domains, DII and DIV together, by means of non-covalent bonds. As seen in Figure 1b, the interdomain-conformational changes within DIII have been so widely moved that the DIII-cluster acidic residues (Asp405, Asp411, Tyr412 and Glu518) on the exposed surface loops lie closely adjacent to the basic helix (Asn236, Lys240, Arg residues 244, 246 and 364) in DII due to Ca^2+^-induced rearrangements of those domains (DI and DII, DIV). Much lower electrostatic interactions in the bovine CAPN1 than in the interaction between the corresponding CAPN2 acidic and basic residues (these residues are highly conserved in Lys residues 226, 230, 234, and Lys residues 354, 355, and 357 on the domain DII’s basic helix across mammalian CAPN2) might be slightly partial negative compensation, but they also yield a more flexible acidic loop (residues 402–417) through many polar contacts. The lack of polar interactions suggests that a structural complementarity may otherwise occupy those positions. Surprisingly, the Asn236 positioned on the basic helix of DII opposing the DIII acidic residues is found only in the bovine CAPN1, whereas all other mammalian CAPN1 possess a Gln236 residue at the same location, which corresponds a residue in CAPN2; almost all mammalian CAPN2 contains the basic residue Lys226, except for a similar basic Arg226 residue found only in the bovine and dog CAPN2. In contrast, the Arg364 residue (the CAPN1 subgroup) and Lys354 residue (CAPN2) always reside in a conserved basic residue within the mammalian CAPN group, possessing a class-specific trace residue at the 364 residue position (Figure 4b). In addition, two Asn residues (365 and 367 corresponding to Lys residues 355 and 357 in CAPN2) are essentially included in the class-specific residues seen in Figure 4b, whereas Asn365 residues always comprise Asn or Lys residues at the position of CAPN1. Essentially, this may be considered a species-specific variation. The Asn variant seems to occur only in mouse, rat, and bovine CAPN1. These basic residues on the DII helix could contribute to a specific “sandwich” structural character and to significant polar contacts of the acidic loop (residues 402–417) in the bovine CAPN1 from the opposite region of the basic loop of the domain (residues 428–438).

Domain DIII itself plays a major role in signaling Ca^2+^-binding to the domains (DI and DII, DIV) and in the Ca^2+^-regulation of enzyme activity. If the electrostatic interaction between the acidic residues and the basic residues of the helix weaken as mentioned above, this would give rise to increased mobility of the catalytic domain DII, which may reduce the requirement of Ca^2+^ ions for assembling the activate protease core form [42,43]. In the presence of Ca^2+^ ions, intradomain-conformational changes in DIII have an active recoiled basic loop in Arg431 and Arg420 from the basic loop (residues 428–438) and Arg483 and Arg500 from the β-sandwich core in DIII with a difficult conformation of the inactive bovine CAPN1 (Figure 1). Even though DIII of bovine CAPN1 has a different topology, the acidic Glu402-Glu417 loop is equipped with a tertiary fold (of β-sandwich) that is very similar to the loop in C2-domains. Here, numerous acidic residues in the loop positions are partially stabilized by adjacent basic residues (Lys398, Arg400, and Arg415). The acidic loop (residues 402–417) within DIII has also been thought to play a role in Ca^2+^-promoted activation of CAPN. Furthermore, it is very interesting that two switch loops with electrostatic properties in DIII are present in opposite directions. The basic loop (Lys428-Arg438) is situated in the CAPN1 molecule center, while the negatively charged loop (Glu402-Glu417) is oppositely located, presenting several acidic residues toward the solvent. This results in stronger electrostatic interactions with the complementary residues of DI and DII, respectively, for Ca^2+^-dependent activation. This may be involved in regulating CAPN activity by participation in critical electrostatic interactions. If replacement of residues reduces their electrostatic potentials in the DIII loops, the release of assembling structural constraints and weaker attraction of DII from DI could be modulated, indicating that the protease domains (DI and DII) are more exposed to the inhibitor binding cleft’s surface, where structural and biochemical constraints are imposed by CAST subdomain B’s physical interactions. As a consequence, either the special features of the site structure, the interaction geometry, or the real interaction sites of CAST’s subdomain B could be changed. In addition, these switch loops (residues 402–417, 428–438) may offer additional membrane binding sites [44], leading to favorable electrostatic interactions and closing contact with the opposite properties of the membrane surface when DIII binds to the membrane. Its high-order structure and amphoteric characteristic are resembled to C2-domains binding to Ca^2+^ ions and phospholipids activation of the enzyme, as observed with protein kinase C and phospholipase C. In particular, the finding suggests that protease kinase A (PKA) prevents EGF-induced, CAPN2 activation for cell migration [6,45] as a structure model of Ser369-phosphorylated CAPN2, in which Ser369 residue in DIII is located in the interaction region between domains DIII and DIV on the other side of the switch loop. The interactive region of the Ser369 residue reacts with residues Arg628 and His643 (corresponding to the interaction region of Ser379 with both Arg643 and Tyr658 residues in bovine CAPN1, respectively); it is almost present in the mammalian CAPN1 and CAPN2 subgroups, but the His643 residue is highly conserved only in CAPN2. In contrast, histidine is replaced by tyrosine at the position of residue 658 among the CAPN1 subgroup. (Conversely, histidine is maintained only in rat and mouse CAPN1, at an identical position.) This stronger polar interaction of phosphorylated-Ser369, along with two key residues, is predicted to rigidify the domain movements between DIII and DIV and hence prevent the formation of an assembling proteolytic core of the enzyme for negative control of CAPN activation [45]. These results are in marked contrast to the relation in mammalian CAPN2.

**Figure 9.**
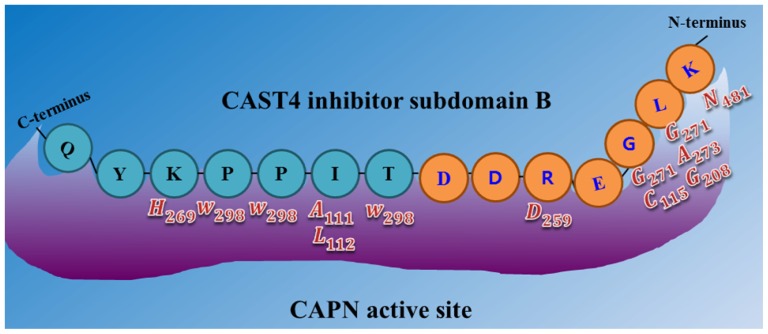
Schematic representation of the CAST4 subdomain B binding to the CAPN active site cleft both from bovines. The fragment *N*-terminal portion is built up of two α-helices and an extended β-strand, which interacts with a surface-localized binding loop of the enzyme. The *C*-terminal segment binds the two enzyme domains being anchored by a short α-helix (after Schechter and Berger nomenclature [24]).

The crystal structures of CAPN2 (PDB code 1DVI for rat, 1NX2 for pig) reveal that domains DIV and DVI are well characterized in the Ca^2+^-binding construction of each domain and contain five EF-hands motif with eight α-helices. Indeed, a comparison of subunits within non-Ca^2+^-bound and the Ca^2+^-bound homodimer of rat CAPN2 [23] reveal neither large overall structural deviations, nor considerable differences in the hydrophobic surfaces exposed to the solvent. As mentioned above, these domains have a high degree of secondary and tertiary structural similarities to each other (sequence identity of 65%–95%) for a CAPN group among the species about themselves. Only the EF-hands motif conformers of DIV and DVI do not seem to be significantly altered by Ca^2+^-binding. Indeed, such conformers undergo similar open-conformational changes on Ca^2+^-loading, corresponding to the ordered EF-hands motif of the inter-heterodimer contact copartner. There is minimal conformational change caused by hydrophobic interactions between the E and F helices of each EF-hand motif and strong helical dipole-dipole interactions in the helix in both the DIV and DVI domains. The local geometry of the EF-hands-motif changes after Ca^2+^-binding, mainly to allow the acidi- residue-side chains (Asp560, Glu562, Glu567 in the bovine Figure 6d) to coordinate the bound-Ca^2+^ ions in agreement with DIV and DVI domain constructions of either type of mammalian CAPN subgroups. Moreover, it can be inferred that the two domains must have similar Ca^2+^-binding patterns (DDXKE, DDSXE, and DN predicted in Table 3) and exposed hydrophobic surfaces (as seen in Figure 9) in the same region, even though DVI was not included in the structural model. The crystal structure of DVI from rat CAPN2 (PDB code 1DVI) indicates that the fifth EF-hand motifs in DIV and DVI interact with each other to form heterodimers comprising catalytic and regulatory subunits through a hydrophobic contact. The hydrophobic interaction interface involves those residues (*i.e*., Leu235, Met241, Phe242, Trp263, Leu264, Met268, and Tyr269) that were identified in the DVI crystal structure (1DVI for rat CAPN2). The putative corresponding residues of DIV in bovine CAPN1 are Leu681, Met687, Phe688, Phe691, Trp709, Leu710, Met714, and Phe715. Interestingly, the same residues with only a few conservative substitutions are also present in corresponding positions of the *C*-terminal of DIV from the catalytic subunit of the CAPN1 subgroup, and these residues are highly conserved across a CAPN group as seen in trace residues at the positions of Figure 4b. This indicates their importance in association with the catalytic and regulatory subunits in both CAPN subgroups. If the regulation subunit is dissociated, DIV of the catalytic subunit exposes a hydrophobic surface and then self-aggregates within the aqueous solvent environment. Those domains have structural characteristics shared by other CAPN subgroup molecules, similar to the conformational change within an active CAPN2 reference molecule (Figure 1b).

In this study, the structural bovine CAPN1 model lacks small regular subunits but has a multi-domain structure very similar to that of the large catalytic subunit of CAPN1 from mammals. In particular, the most remarkable differences in that the *N*-terminal anchor construct is displaced, resulting in the formation of the functional protease domain originating from its Ca^2+^-dependent activation, as is well known in mammal CAPN structures. Reference structures are presented in Table 4. The DVI structure in the DIV/DVI heterodimer is almost a duplicate of its structural homodimer as DIV/DIV (r.m.s.d. of 1.29Å on main chain atoms [20]). Furthermore, many CAPN studies [20,22,23,35] have provided support for the idea that the interactions between the anchor helix and DVI are equivalently identical in a CAPN1/CAPN2 chimera (PDB code 1KXR) and the wild-type CAPN2 (PDB code 1DF0). We are very confident that the model structure will be sufficient to apply to defining interactions between intra-inter domains provided for cooperation and to provide explanations of the relationships between structure and function relationships for Ca^2+^-signaling in bovine CAPN1. Fortunately, based on alignments between sequences and structures as well as analysis of calcium-binding patterns, the structural information contained in our bovine CAPN1 model, together with prediction of interaction site mutation effects on the enzyme stability, binding affinity to Ca^2+^ ion or subdomains AB of CAST, and self-aggregation should facilitate the design and virtual screening of inhibitors that target mammalian CAPN1.

### 2.2. A Structural Model for the Inhibition of CAPN1 by CAST4 from Both Bovines

#### 2.2.1. Structure Modeling and Evaluation of the Bovine CAPN1/CAST4 Complex

We want to use the CAPN crystal structures that have inhibitors bound in their active sites to develop a better model, that is, one that may better predict the potential of the inhibitory qualities of small molecules as well as the differential selectivity between the CAPN subgroups. However, CAPN1 and CAPN2 are very similar in the construction of their active site; thus, most inhibitors of one form inhibit the isoforms of the other CAPNs. This is supported by the finding that the two CAPN subgroups share nearly identical CAPN substrate preferences [14,15,46,47]. Despite the physiological importance of these interactions, the basis of constraint on the interaction for CAPN absolute specificity and binding selectivity in CAPN1 inhibition by CAST is not known from any structural information of the regulation system. To determine whether this structural similarity entails functional analogy, we have characterized the constraints imposed by domain interactions from the bovine CAPN1/CAST4 system. Although there is no X-ray crystalline structure of an active CAPN1/CAST4 complex, both sides of the binding pocket of CAST4 subdomain B are thought to be involved in the domains DI to DIII. In particular, the communicated interaction interface in DIII of those domains might have selectivity of the enzyme’s substrates among various CAPN isoforms. We were previously able to highlight the significant effect of the non-synonymous SNPs (two CAPN1 variants: Gla316Ala and, Val530Ile and, one CAST4 variant: Ser649Thr) on the tenderness and flavor of Hanwoo (Korean cattle) by molecular modeling of the bovine CAPN1/CAST4 system. The molecular modeling of the CAPN530 (Val530Ile) mutation may show stronger inhibition of the CAPN1 enzyme by CAST4 protein binding, resulting increased toughness in Hanwoo [48]. The model structures should provide new opportunities for insights into SNP markers highly related to carcass characteristics (meat quality) for bovine CAPN1. To further generalize and improve the structural knowledge base of CAPN1/CAST4 interactions, this study focused more on the relationships between structure and function of the regulatory system. In particular, this study focused on whether the model structures reflected how changes in the constraint at the interaction sites could affect either CAPN1 itself, or its complex with CAST4, using virtual residue mutations.

The bovine CAST is encoded by a single gene, which exists on chromosome seven. This gene produces a number of closely related protein isoforms via alternative splicing, which is most likely the cause for the molecular diversity [49,50]. The protein contains one non-homologous sequence on the *N*-terminal side (domain L) and four repeating inhibition domains (domains 1–4; CAST1-4 corresponding the inhibitory domains are present in residues 167–294 for CAST1, residues 302–429 for CAST2, residues 440–572 for CAST3 and residues 583–710 for CAST4 from bovine). Each of these CAST domains is capable of binding to CAPN, such that a single CAST can inhibit several CAPN molecules; however, its *N*-terminal L domain alone has no inhibitory activity. Three highly conserved regions within each repeat domain comprise three subdomains A, B, and C. When CAST has a complex interaction with CAPN, the A subdomain interacts with DIV, and the C subdomain bonds with CAPN DVI. The B subdomain inhibits CAPN by contact with its protease core (DI and DII) and cross-links to DIII [8]. Each CAST domain can independently interact with different domains of CAPN through similar inhibitory activity of almost subnanomolar affinity, but only in the presence of Ca^2+^ ions at the concentrations required for *in vitro* activity (~10–50 μM in CAPN1, and ~300–500 μM in CAPN2 [51,52]). The relative binding affinity of each inhibitory domain for CAPN2 varies from most to least effective as follows: CAST1 > CAST4 > CAST3 > CAST2 in its endogenous inhibitor CAST, but the ability to inhibit two CAPN subgroups is not consistent among the different CAST domains [35,41,53]. Surprisingly, the CAPN1-catalytic domains (DI and DII) are not inhibited by CAST1 or by a peptide corresponding to a segment of subdomain B in isolation, which possesses CAPN-inhibitory activity [35,54]. These inhibitory profiles suggest that CAST does not bind only at the protease core of CAPN. This is further, supported by the differences in the inhibition’s specificity between CAST subdomains including some differences in the binding site’s specificity and its binding capacity between a partial (domains DI and DII; mini-calpains as surrogates for the whole enzyme) and full length CAPN. That is the domains (DIII-DIV) of other catalytic subunits of the full-length CAPN can have some impact on the binding specificity of the CAST4 subdomain-B segment. Perhaps most importantly, all prominent contacts are mediated through the flexible portion of the polypeptide chain within CAST4 subdomain B, residing close to both the active site and an as yet unidentified site with domain DIII of CAPN, whereas the CAST subdomains A and C do not contribute directly to protease inhibition, which may be responsible for the stabilization of the inhibitory complex. This results in an increase in the overall affinity of the interaction. Another possibility is that the individual CAST subdomains may have different affinities for tissue-specific CAPN isomers.

For bovine CAST, the order of the sequence identity of each inhibitory domain compared with the corresponding domain of mammalian CAST is variable among the different CAST domains. In particular, the sequence homology of CAST1 and CAST4, which strongly inhibit proteolytic activity among species, is less similar than for other domains (from 51% to 76% and from 59% to 78%, respectively) In contrast, CAST2 and CAST3 have more than 65% sequence identity across species. In addition, the amino acid composition of skeletal muscle CAST and that of cardiac CAST from the same species of bovine, presents substantial differences [50,55] as the calculated isoelectric point (pI) of 4.75 for the former and a range of 4.85 to 4.95 obtained by the method of Skoog *et al*. [56]. The specific character also allows distribution of charged residues in human CAST domains 1–4 (pI 4.26–4.90) to be acidic, in contrast to domain L, which is basic (pI 10.23) [57]. In other words, the tissue specificity of multiple isoforms of the CAST in bovine tissue is displayed by various patterns; the CAST domains can differ significantly in their ability to inhibit CAPN1 or CAPN2 and in the relative specificity for both them according to the subdomains (A, B, and C) of each inhibitory domain.

We predicted the active complex structure between the catalytic subunit of CAPN1 and the partial regions of CAST4 (residue 638–696) from bovines. In particular, owing to the lack of CAST sequence homology with any known protein, we performed a comparative modeling using only the template structure 3BOW of the *C* chain, which is rat CAST4. The modeled structure of CAST4 is composed of subdomains AB. Therein, CAST4 subdomain A folds as an acidic amphiphatic helix bound to a Ca^2+^-induced hydrophobic pocket in DIV. The subdomain B architecture extends from a two-turn amphiphatic helix on the *C*-terminal side to an extended polypeptide on the *N*-terminal side, covering the surface of CAPN1 and putting it in contact with multiple-domains from DI to DIII in the bovine CAPN1. The statistical values reflecting the quality of the model structures of the Ca^2+^-bound, bovine CAPN1/CAST4 complex are presented in Table 2. The complex model structure between subdomains A and B of CAST4, and a larger catalytic subunit (DI-DIV) of CAPN1, has similar quality and overall structural frames that are similar to its template structure (rat CAPN2/CAST4 domain complex-3BOW). The model structure of the modeled complex shows that 89.0% of the residues were in the most favored region and 9.5% in the additionally allowed region of the Ramachandran plot. The corresponding crystal structure of 3BOW is 88.9% and 9.5%, respectively. Almost 0.9% of the residues of our model and 1.2% of the template structure (3BOW) were in generously allowed regions of the plot, and coincident residues in disallowed regions were 0.6% (model) and 0.4% (template). (The plot is not shown for the complex structure). Figure 3 also presents a parallel of the density plot between the predicted model of the complex and a template structure (3BOW). Compared for each reference structure, the bovine CAPN1/CAST4 complex model has a QMEAN score of 0.67 and differs from 641 reference structures consisting of about 740 residues (±10%) with a standard deviation of −1.04, while the corresponding template structure (3BOW) has a QMEAN score of 0.68 and a standard deviation of −0.82 with 919 residues (±10%) for 414 reference structures. The enzyme and the model of its inhibitor complex could be accommodated without large disruption of the overall binding structure; therefore, the predictive complex model is suitable for examination of the individual contributions of each key residue to the overall inhibitory activity.

#### 2.2.2. Functional Characterization of the Inhibitory Motif of CAST4 Subdomain B

CAPN is recognized by a specific inhibitor protein (CAST) after activation by Ca^2+^ ions, which bind to its active sites while remaining uncleaved unlike its substrates at the same binding sites into inhibiting the enzyme activity. Then CAST may be considered a poor candidate for cleavage while providing insight into determination of the enzyme’s substrate specificity. Knowledge of the relative contributions of the side chain and backbone functionalities of each key residue to the overall CAPN binding affinity is required for determining, within constraints on interaction, how CAST interacts with CAPN to inhibit its enzymatic activity. Interaction constraints exist over wide contact regions [58] of the CAPN/CAST system, where both the enzyme and its inhibitor are under strict Ca^2+^ ion control. An interesting point is that the conserved inhibitory sequence (TIPPXYR motif) of the mammalian CAST4 subdomain B shows very similarly positioned residue preferences fitted to the active site of the ideal of CAPN substrate on the *C*-terminal side of the cleavage site [15,47]. This supports the notion that CAST subdomain B can bind to the binding pocket of the equivalent substrate as a substrate analog by competitive inhibition of the enzyme. Using a reversible approach, the binding constraints of these sites concerning CAST action can also be rationalized via this similarity. The active enzyme sites can accommodate various residue-residue interactions within their inhibitor-binding contacts. Regarding the binding constraints of CAST4 to CAPN1, it the following facts were recognized early. (1) Effective inhibition by CAST requires that all subdomains (A, B, and C) simultaneously bind to CAPN at three sites on the enzyme molecule; (2) In the residues of each subdomain among the four repeat domains (from CAST1 to CAST4), the 14 amino acid residues in subdomains A (XDXALXXLXXSLGX sequence) and C (XXDPXDALSXDXDS sequence) were remarkably conserved among the mammalian of CAST molecule bound to domain DIV and DVI of CAPN, respectively, while the conserved 12 residues (GXXXXTXPXXYR sequence) in subdomain B binding to an area near the active site of CAPN are essential for the CAST inhibitory activity [59,60]. (Interestingly, whether it has species or tissue specificity is not clear); (3) The numbers of amino acid residues between subdomains A and B and between subdomains B and C in bovine cardiac CAST4 are almost the same (29 and 30, respectively) [49]. Indeed, the distance between the binding sites of bovine CAST4 in domain DIV and DVI and the Ca^2+^-induced catalytic site in DI and DII may be the same as the distances between subdomains A and B and between subdomains B and C for the crystal structure (3BOW) in the rat CAPN2/CAST4 system. Thus so, it should be possible for all three subdomains to bind to the CAPN molecule at the same time, but only in the presence of Ca^2+^ ions for maximum inhibitory efficiency [49,53]. These specific characteristics of CAST’s observed inhibition of CAPN have revealed that the inhibitor’s binding modes, such as key residue accessibility, backbone conformation, or three-dimensional structure, are also consequential factors with the specificity profile for residues found at counterpart’s positions within the regulation system in the inhibitory activity determination. Indeed, this relative binding mode appears to be remarkably well conserved even in the modeled bovine CAPN1/CAST4 structure.

In the model structure, it was immediately obvious from the template structure (rat CAPN2 from 3BOW) that, for bovine CAPN1 catalytic subunits, although they already have similar interaction contacts for nonspecific binding to the enzyme substrate and inhibitor, class-specific interaction constraints must exist in the enzyme binding pocket. As expected from Table 4, both regulation systems share fundamental biochemical properties in their interaction constraints. The modeled bovine catalytic subunit (DI-DIV) shares approximately 60% sequence identity with rat CAPN2 and 73% sequence identity of the CAST4 subdomains A and B from both these species, and highly conserved residues (The LDDALDXLSDSLGQ motif [59], GERDDTIPPXR motif [61] in subdomains A and B derived from CAST4 are capable of inhibiting CAPN, respectively), which are critical for inhibitory function within subdomains AB of CAST4 both mammalian species. It should also be noted that most of the interaction constraints between CAST4 subdomain B and a complete set of active site residues (Cys115, His272, Asn296) along with other critical flanking residues (Pro297, Trp298) on the CAPN1 protease core, were highly conserved among CAPN homologs. Even though the whole CAPN1/CAST4 complex structure has not yet been predicted in the presence of Ca^2+^ ions, the observation can further be explained by the fact that bovine CAPN1 and rat CAPN2 show great structural homology in their Ca^2+^-bound conformations (Figure 1b) thus, contributing to the preference for similar interaction sites against their inhibitor domain, CAST4. No the interaction constraints on CAST4 would have provided a dramatically different in which to function. Instead, both the active sites may significantly differ in their effects on the overall strength of interaction within the enzyme/inhibitor system. The conserved interaction interfaces (e.g., cysteine protease) could be clustered in several critical interaction constraints shared by CAPN1 and CAPN2 subgroups across the species. Some constraints lead to residue replacement affecting the physical interaction with CAST4 and its binding affinity. Such sites are considered to accentuate the specificities modulating interactions of CAPN with other proteins at the cleft of the active site [13–15,61]. We have attempted to further interpret these constraints in accordance with former significant trace-residues of both of the CAPN subgroups necessary to bring the enzyme to its functional importance and evolutionary conservation by all key residue-residue interactions on the contact interfaces between mammalian CAPN1 and CAST4. The trace residue characteristics of the different mammalian CAPN subgroups based on their structural alignment (in Figure 4b) reflect their functional and biochemical similarities. Thus, the constraints on most of these site were generally conserved in equivalent biochemical properties of the corresponding residue in bovine CAPN1 and other mammalian CAPN1 and CAPN2 subgroups that could not directly be related to interaction specificities distinguishing the bovine CAPN1 itself, from other members of the CAPN group. However, apart from CAPN2, the interaction specificities of bovine CAPN1 alone could be identified in the class-specific trace residues split into active site residues and potential substrate binding cleft within a mammalian CAPN1 phylogeny. Knowledge about such species- and class-specific trace residues could support the investigation of interaction constraints in the regulation system of bovine CAPN1. Preferentially, we explored how constraints on selective interaction varied if the trace residue’s replacements could modulate the strength of interaction of bovine CAPN1 at the contact regions with CAST4 by considering their biochemical and functional properties. The CAST4 subdomain A and B binding sites to the CAPN1 molecule must be involved a wide range of interaction sites to recognize their substrates rather than binding strongly to a few specific residues around the cleft in the catalytic core. When we mapped the bovine CAPN1/CAST4 complex model to a template structure (3BOW), it revealed some biochemical constraints different from those previously observed for both CAPN subgroups on the basis of the rat CAPN2/CAST4 complex. The specificity of CAST4 subdomain B to the bovine CAPN1 was seen in several adjacent hydrophilic residues Lys678-Asp684 (highly conserved KLGERDD peptide sequence in mammals) looping out from the protease core cleft into specific and effective inhibition of CAPN1, and on the other hand, critical residues (TIPPEYR peptide sequence in mammals) fitted to the active site into the enzyme activity to control multiple aspects of their interactions with each other (Figure 9). Most notably, the conserved TIPPEYR sequence in CAST4 subdomain B bound to the protease core of CAPN1 was previously identified as an important residue across mammals for inhibitory activity [15,47]; however, on the *C*-terminal side of the bovine CAST4 subdomain B, it corresponds to the TIPPKYQ sequence (residues 685–691). In addition, the Leu679 and Gly680 residues of the CAST4 subdomain B are known to be essential for enzyme inhibition. The side chains of these residues make key hydrophobic contact with the Cys115 residue, as one of the catalytic triad, as well as its surrounding residues (Gly208, Gly271, and Ala273) in the catalytic cleft of bovine CAPN1. These hydrophobic contacts are more tightly mediated by the formation of H-bonds between the backbone atoms of the residues (Leu679 and Gly680) in the inhibitor subdomain B, and on the side chains of Gly208 and Gly271 in the catalytic cleft. On the other hand, the formation of a β-turn structure between Leu679 and Gly680 residues prevent its peptide bond’s cleavage of glycine from the catalytic Cys115 residue’s nucleophilic attack. Moreover, the highly conserved Ile686, Pro687, and Pro688 residues in the TIPPKYQ sequence make favorable hydrophobic contact with both side-chains of the Ala111 and Leu112 residues in bovine CAPN1 when the two proline residues (Pro687 and Pro688) provide π-stacking interaction with both side chains. Trp298 in DII contributes to increase the hydrophobic moment at the interaction interface (in Figures 8b and 9). At the same time, the other key residue (Thr685) is hydrogen-bonding with Trp298, which, thus, maintains a functional local conformer at the interaction site. As another factor, the stability of the electrostatic interaction between the glycine rich region (Gly207–Gly220 residues) in DI and domain DIII without pragmatism Trp116 residue would add to the interaction constraint as position-based features for substrate binding and catalysis with Cys115 and Gln109 residues. The remainder of the subdomain-B interaction-residues are summarized in Table 5, and they are compared to the template of the rat CAPN2/CAST4 complex (PDB code: 3BOW).

## 3. Discussion

**Figure 10.**
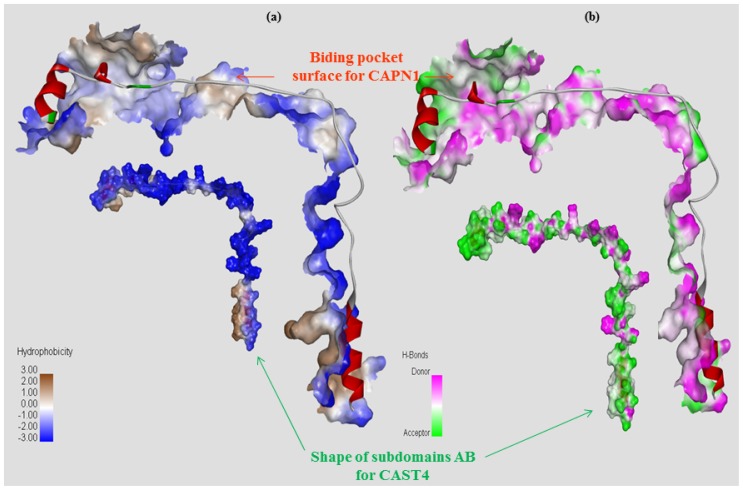
The bovine CAPN surface atoms that are close to the current CAST4 subdomain B: colored (**a**) by hydrophobicity of the enzyme interaction residues: hydrophilic (blue), hydrophobic (brown) and (**b**) by hydrogen bond character: receptor donors (green) and receptor accepters (cyan).

In addition to the conserved intermolecular interactions seen in Figure 9, another contributing factor may be the narrowness of the binding pocket around the active nucleophilic site (Cys115 residue). When subdomain B binds to the activated enzyme on either side of the active site cleft, the conserved *C*-terminal regions (the TIPPKYQ sequence) have two-turn *α*-helix structures (the Lys689-Asp696 helix) buried inside the narrow cleft, bordered by two smooth-faced sides. The sides are made up of domains DI and DII, and the direction of the helix is induced from the PPKY sequence’s backbone conformer (residues 687–690) targeted to the domain DI. The *N*-terminal side of CAST4 subdomain B engages an area exposed at the surfaces of CAPN DII to DIII, permitting direct interaction with the corresponding residues. This further potentiates the inhibitor activity of the CAST4 domain. DIII, with a shallow serial groove, also contains extensive hydrophobic and electrostatic interactions with CAST4 subdomain B in the complex system (Figure 10). Bovine DIII is also important for binding its inhibitor with more than 27 residues of CAST4 subdomain B in contact with them, as seen in Table 5. There is a significant specificity difference across either type of CAPN subgroup, as shown in Table 5, with CAPN1 class-specific trace residues in blue and the only bovine species-specific trace residues in red. In particular, these interaction residues are also involved in the electrostatic switch loops and are points in a different direction contact with CAST4 subdomain B. The comparison in Table 5 shows that the two CAPN isoforms display broad specificity toward the CAST4 subdomain B; this is consistent with the idea that CAPN group members presumably share an analogical mode of inhibition by CAST4. Indeed, two clustered key residues (Leu11–Gly12 and Thr17-Ile18-Pro19) in B27-WT [47] (a 27 residue peptide derived from human CAST1 subdomain B), show strong inhibition against two CAPN isoforms, within which the residues critical for CAPN inhibition against bovine CAPN1 result in no obvious difference between their profile and that of rat CAPN2 (template structure 3BOW) proceeding through direct interactions with the catalytic center Cys115 residues and surface residues (Gly271, Ala273, Gly208, Trp298, Ala111, L112) adjacent to the active center of CAPN for ligand-receptor interactions as illustrated in Figure 9. However, different overall interaction constraints were reflected by additional contacts with the surface of the enzyme domains DI-DIII, determining the high specificity of the bovine CAPN1 and discriminating them from the relatively non-specific cysteine proteases. On the constraints imposed by the cleft-size of active site that act through steric blockage of the subdomain B fragments access to the enzyme catalytic center resulted in the same relative preferences of positioned-residue in all of the protease core highly conserved CAPN subgroups. A greater difference in specificity is observed with most of the key contact residues, comparing class-specific and bovine specific trace residues not only concentrated on the less similar domains (DII and DIII) rather than others (DIV and DI) across the mammalian CAPN group, but also predominantly lie in the exposed interaction interface (Table 5 and Figure 5). These positions are far enough from the active site (Cys115) residue to shift the center of major interaction contacts toward the DII/DIII interface. The additional binding affinity may be influenced by other catalytic domains in the whole enzyme. This reflects the importance of species-specific and class-specific variations in key residues involved in substrate and inhibitor recognition apparent from these interaction profiles.

The conserved hydrophobic residues (Leu639, 643, 646, 650, Ala642 on the bovine CAST4 residues 639–652 of LDDALDQLSDSLGQ motif) of subdomain A engage deeply into the hydrophobic pocket between the EF-hand 1 (residues 543–587) and 2 (residues 587–620) in DIV. The CAST4 subdomain A, bound to the DIV hydrophobic pocket, is shown using the hydrophobicity scales of the enzyme interaction residues (Figure 10a). In the overall structural homology of the template structure of rat CAPN2/CAST4, the topology of the subdomain A binding region in our model was kept along with a structural model for the complex between domain DIV and the DIA19 peptide (a 19 residue peptide corresponds to subdomain A of pig CAST1) of CAST provided by Todd *et al.* [18]. The trace residues in the large part of special bovine CAPN1 are lined with a hydrophilic surface of between EF-hands motif 1–2 that interact with the neighboring polar residues of the amphipathic helix of subdomain A, while the hydrophobic trace residues are inside the binding-site groove that provides an even larger hydrophobic surface in the same region (Table 5 and Figure 10). The bovine trace residues present on the binding site of domain DIV show no pronounced interactional preferences even in the highly conserved binding modes. In both structures of the active site, on the binding pocket with an inhibitor, the interaction constraint clusters are located at domain/domain contact interfaces. Of these, the main contacts with CAST4 subdomains A and B are also associated with the positions of the LGMD-2A mutations [40]. The loss of CAPN3 activity is responsible for disease. These missense mutants (corresponding mutations in the bovine CAPN1: Arg104Gly, Glu203Lys, Gly208Arg, Glu212Lys, Arg385His, Asp600Gly) had specific activities consistent with a pathogenic role in LGMD-2A. All are found in the corresponding positions in highly conserved residues between two mammalian CAPN subgroups (Figure 4b).

## 4. Materials and Methods

### 4.1. A Structural Model of the Bovine CAPN1 Protein in the Absence of Calcium

CAPN1 crystal structures are known only in the Ca^2+^-bound cysteine protease core (DI and DII) and a CAST inhibitory domain (DIV/DVI) in mammals, including rats and humans (PDB code 1QXP, 1KXR and 2ARY). These domains are parts of a homologous catalytic subunit containing four domains (DI through DIV) and a common regulatory subunit containing two domains (DV and DVI). The protein sequence of bovine CAPN1 is very similar to those of rats and humans (overall sequence identity is above 90%), of which the partial structures are already known. This protein structure can be predicted based on the assumption that unknown bovine CAPN1 is similar to the known structures of some homologous reference proteins. These were therefore of considerable usefulness as templates to determine and compare the active and inactive bovine CAPN1 structures in order to understand the relationship between structure and function of the bovine CAPN1 and CAST 4 domain (CAST4) complex. To build homology models for the 3D structure of the enzyme from its protein sequence, the alignment of the bovine CAPN1 sequence with the template sequences was achieved by creating a sequence profile using the SwissProt protein database (http://www.expasy.org) BLAST search (http://web.expasy.org/blast/) and then aligning the sequence profile to the pre-aligned template structures using structure alignment. Following sequence alignment, the coordinates of the reference proteins were then used to predict those of bovine CAPN1. It seems likely that the conformational similarities between templates are more important than the sequence similarity between the model sequence and the templates. If the templates all have homologies similar to the bovine CAPN1 sequence and have high structural similarity at the core, they may still be different from each other (e.g., different lengths for some loops and different loop conformations). As sequence conservation and structure conservation are often different, the best template is one that has high structure similarity, covers the entire sequence length, has a fairly high sequence identity, and has a good *E*-value (<1 × 10^−5^). The high-resolution structure of the template is also an absolute requirement, because the structural features of the reference proteins are used to set spatial restraints that serve as the basis for optimization of the model. Table 4 shows several reference proteins used in the homology model building process.

**Figure 11.**
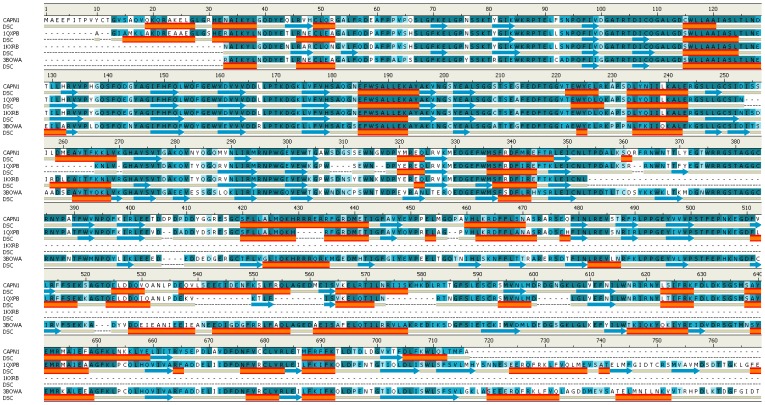
Sequence alignment between the bovine CAPN1 and templates (identity: 59.4%–80.2%; similarity: 77.0%–82.4% with templates (1QXP:B, 1KXR:B, and 3BOW:A) and secondary structures(alpha helices: red bars; beta strands: blue arrows; coil: beige bar) based on sequence profile.

Thus, the bovine CAPN1 protein sequence to template structures (PDB code 1QXP:B, 1KXR:B, and 3BOW:A) was mapped to the conserved core by the sequence identity 59.4%–80.2% and similarity 77.0%–85.8% between them (Table 4 and Figure 11).

The structural model of bovine CAPN1 (from Tyr10 to Ala716 residues) was then created using the MODELER9 v8 program within the Discovery Studio interface. The structural model generated with MODELER [62] (Sali *et al*., 1995) had loop regions (Ser255-Met260, Ala306-Ser312, Ser360-Tyr371, Leu540-Phe549, and Arg574-Leu582) present in the bovine CAPN1 sequence, which were not aligned to any template structures. Therefore, we refined the loop conformation using the discrete optimized protein energy (DOPE) scoring function (Shen *et al*., 2006) [63], which used by loop optimization in the MODELER loop refinement as a measure of model quality. The energy function included bonded terms (for example, bond length, bond angle, main-chain dihedral angle, side chain dihedral angles, and so forth) and non-bonded terms (loop non-bonded terms of DOPE; Lennard-Jones, DOPE statistical potential, charge-charge interaction, and electrostatic contribution to the solvation energy calculated by a Generalized Born implicit solvent model). The DOPE-loop-potential function measures the relative stability of a conformation with respect to other conformations of the same enzyme. The model with the lower score is the better one. The side chain conformation of the enzyme is afterwards optimized through a process of systematic searching for side chain conformation, and by CHARMm (chemistry at Harvard molecular mechanics, CHARMM, its commercial CHARMm version distributed by Accelrys, San Diego, CA, USA ) energy minimization. Following the loop refinements, the optimization of generated model structures take place in two distinct stages; the first is conjugate gradient optimization and the second optimization stage consists of a restrained molecular dynamics (MD) with simulated annealing scheme for a model protein structure. This approach first satisfies sequentially local restraints and slowly introduces long range restraints defined in terms of the geometric features in reference structures, until the complete objective function (F), that is the natural logarithm of the molecular PDF (total energy probability density functions) [64], is optimized by MODELER program within the Discovery Studio interface.

An assessment of the 3D structure model of the final bovine CAPN1 (Figure 1a) involved two independent tests evaluating its internal stability and reliability. The first was a PDF test, describing the geometric features of the molecule, such as bond length, bond angles, dihedral angles, and non-bond distances. The PDFs are derived statistically and empirically from a database of known protein structures that set the spatial restraints derived from the stereo-chemical and homology of the templates. Higher PDF total energy indicates a larger restraint violation in the model; therefore, lower score indicate a better model. The second test was carried out using QMEAN score6 [31] on the SWISS-model Workspace assessable via the ExPASy web server (http://swissmodel.expasy.org/) This QMEAN [31] score is a score reflecting the reliability of the predictive model in a range from 0 to 1, with higher values indicating more reliable candidates. This score is able to drive both errors of the entire structure and per residue, on the basis of one predictive model. Taken together the QMEAN *Z*-core is an estimate of the comparable quality of a model as the native degree of the structural features in the model by describing the likelihood of the model to reference structures solved by X-ray crystallography.

### 4.2. Predicting the Complex Structure between Calcium Bound, Bovine CAPN1 and CAST

The endogenous CAPN inhibitor CAST is an unstructured protein for which it is possible to reversibly bind four molecules of active CAPN at the same time. CAST is well conserved with greater than 65% protein sequence identity across mammalian species, but with no homologs from any proteins sequenced, thus, far. To date, the only know complex structures for Ca^2+^-binding to CAPN2/CAST4 or to the CAPN2/CAST1 proteolytic system are from rats (PDB code3BOW, 3DF0), while the complex structure of the CAPN1/CAST system is not known in other species, including other mammals. The Ca^2+^-binding to CAPN is a prerequisite for their interactions that allows CAST to recognize only the Ca^2+^-induced conformation of CAPN in order to inhibit it. We attempted to predict the interaction process in Ca^2+^-induced binding of CAPN1 to CAST using the model structures.

These regions (from Asn33 to Leu353 of the bovine CAPN1) specify which coordinates are threading from a specific template (PDB code 1KXR:B), and one Ca^2+^ ion is bound within each of the DI and DII domains at the same time. If there are insertions in the bovine CAPN1 with respect to the template, those regions (Ser255-Met260 and, Asn283-Val289) are defined as a loop, and loop optimization and refinement of the side chain in these regions are achieved in the built model. To consider the effect of inter-conformational changes and structural realignment of the other regions (DIII, DIV) during enzyme activation, the model structure was merged by substructure alignment based on the template’s structural similarities (PDB code 1QXP:B and 3BOW:A). In the merge process, one Ca^2+^-binding to the EF-hand added more the DIV. The generated model of an activated bovine CAPN1 was further optimized by restrained molecular dynamics and a simulated annealing process in the whole region (from Ala34 to Ala716, Figure 1b).

Apart from building the activated bovine CAPN1 model, another homology model allowed us to build a bovine CAST substructure (Glu638-Asp696) based on pair alignment of its CAST sequence with the only template 3BOW of the C chain (3BOW:C) on rat CAST4 (Glu571-Asp661, 95 residues). Since the CAST protein sequence is unique, it has no sequence homologous to any polypeptide that has been sequenced before. When compared with each other, the subregions of bovine CAST show sequence conservation of 72.9% identity and 86.4% similarity, to those of the rat CAST4 (Glu571-Asn629) as seen in Table 4.

The conserved residues may be essential for maintaining the CAPN enzyme function, but class-specific residues are responsible for the functional specificity in subgroups (CAPN1 or CAPN2). First of all, we analyzed a specified set of residues, which included both conserved residues and class-specific residues extracted from a multiple sequence alignment. To these were added some other reference proteins known as active CAPN structures (PDB code 1KFU:L, 1KFX:L, 1DF0:A, 1U5I:A, and 3DF0:A in Table 4) with their whole sequence or their protease core regions (Figure 4). In Figure 4, the conserved residues are defined as residues conserved across a CAPN group, and class-specific residues are defined as residues conserved within each subgroup (CAPN1 and CAPN2) but different between subgroups.

In the structural model of bovine CAPN1 inhibition by CAST, the initial interaction locations of the CAST subregions are specified according to CAST positions of the template (PDB code 3BOW:C). The docked complexes are superimposed in such ways that they induce Ca^2+^ to activate the CAPN1 structure, and three Ca^2+^-binding sites of the template (PDB code 3BOW) as seen in Figure 1b. This allows soft body docking to two proteins on the interaction interfaces, where conserved residues, buried residues, and Ca^2+^-binding sites were considered as immobilized locations, while the other class-specific residues were considered flexible. A CHARMm-based refinement procedure and restrained molecular dynamics have been reapplied to optimize the locations of side chains for the specified residues set in interaction sites of the bovine CAPN1/subdomains AB of the CAST4 complex. Then the solvent effects were considered using an empirical scoring function as dielectric constant value 80 (as water environment). In the process, the coordinates of all the hydrogen atoms and structural optimization of the entire hydrogen-bond network were followed by the calculation of electrostatic effects at pH 7.4 in the enzyme molecule because the enzyme itself exhibited optimal, Ca^2+^-dependent proteolytic activity at the natural pH (as we can see from its name of calcium-activated natural protease CANP). Homology modeling and all computational studies were performed with the Discovery studio (DS) 3.1 molecular modeling package [65] on a personal workstation.

## 5. Conclusions

We presented a model of bovine CAPN1 in which it undergoes considerable and important changes in its conformation upon Ca^2+^-binding; further changes occur in the exact interaction patterns of the contact regions observed among trace residues when the inhibitor CAST binds. Even though the enzyme catalytic center (Cys115, His272, Asn296) in the protease core shows highly conserved residues across mammal species as well as the finding of similar motif of CAST4 subdomain B (GERDDTIPPKYQ motif in the bovine CAST4), we were motivated to explore the difference of recognition specificity between CAPN1 and CAPN2 by the inhibitor. In the presence of Ca^2+^, the binding of the subdomain B motif to CAPN1 does not seem to proceed through direct interaction with the catalytic center Cys115 of the protease; rather a part of the inhibitor motif (the TIPPKYQ sequence) binds adjacent to the enzyme active site center that interacts with the key hydrophobic contact residues (the residues Trp298, Ala111, Leu112 of CAPN1). The entire CAST4 subdomain B is seen to inhibit the bovine CAPN1 by occupying both multiple affinity sites present as an extended polypeptide both in and around its active site cleft over the surface from its domain DI to DIII making contact with each domain of the only activate enzyme conformers. This can be valuable for determining any structural feature (the amphiphilic α-helical motif) of binding that may be essential for CAST4 subdomain B to be effective at binding the enzyme, and the interaction patterns are intended to reflect the importance of these corresponding positions in generating specificity for the bovine CAPN1. In conclusion, structural studies with the predicted complex model might reveal differences in its size and depth as well as the properties between the active site of the two protease subgroups (CAPN1 and CAPN2), and this could have some impact on the distinct differences in their specificity against the counterpart CAST. While this study was able to predict how CAST subdomains A and B interact with bovine CAPN1 to elicit an inhibitory effect, more investigation is needed on the CAPN1/CAST variant complex or the variant alone has affecting the extent to the proteolysis activity and searching virtual screening candidate small molecules inhibitors based on protein structure-function in the regulated system. The final reliable models have been deposited in the Protein Model Database (http://mi.caspur.it/PMDB) [66] and have been accessible to the public (PMDB ID: PM0079218, PM0079221, and PM0079222 in both inactive and active model structures of the bovine CAPN1 and in the Ca^2+^-bounded complex of the two subdomains CAST4, respectively).

## Figures and Tables

**Table 1.** Protein sequence identity among mammalian species CAPN1 large subunits (DI through DIV) and full sequence of CAPN1.

CAPN1 *vs.* Bovine	Sequence identity (%)				

	Domain I (DI)	Domain II (DII)	Domain III (DIII)	Domain IV (DIV)	Overall *
House mouse	91.6	85.5	87.3	93.6	89.5
Norway rat	91.6	84.9	87.3	93.6	89.7
Dog	95.3	91.0	94.3	97.7	94.7
Rhesus monkey	94.8	93.4	93.6	98.3	95.1
Human	95.3	91.6	94.3	98.3	94.7
Chimpanzee	95.3	92.2	94.3	98.3	94.8
Bovine CAPN2	77.0	65.7	59.9	51.7	62.5

* Overall amino acid sequence identities (%) are shown, as are those for each domain for CAPN catalytic subunits.

Bold letters indicate a compared to between two calpain isozymes (CAPN1 and CAPN2) from bovine through four domains.

**Table 2.** Estimating the quality of protein structural models in order to rank them.

QMEAN6 score	Inactive template structure (1QXP:B)		Inactive bovine CAPN1 structural model		Activated template structure (1KXR:B)		Activated bovine CAPN1 structural model		CAST4 bound to CAPN2 of template structure (3BOW)		CAST4 bound to CAPN1 structural model	

	Raw score	*Z*-score	Raw score	*Z*-score	Raw score	*Z*-score	Raw score	*Z*-score	Raw score	*Z*-score	Raw score	*Z*-score
C-beta interaction energy	−170.90	−0.82	−124.35	−1.00	−73.49	−1.20	−160.43	−0.63	−314.42	−0.01	−213.98	−0.30
All-atom pairwise energy	−16,945.12	−0.41	−14,017.57	−0.85	−7440.68	−0.89	−17,049.35	−0.18	−30,398.27	0.40	−19,083.34	−0.46
Solvation energy	−31.92	−1.62	−27.92	−1.65	−11.59	−2.08	−36.17	−1.21	−110.00	0.60	−52.80	−1.30
Torsion angle energy	−69.46	−2.81	−79.90	−2.55	−111.55	1.03	−179.04	−0.24	−230.02	−0.34	−190.68	−0.27
Secondary structure agreement	78.2%	−0.12	80.2%	0.28	74.8%	−1.40	80.5%	0.35	80.4%	−0.19	78.1%	−0.76
Solvent accessibility agreement	74.7%	−1.16	74.9%	−1.13	89.4%	1.64	78.2%	−0.54	76.6%	−0.58	75.9%	−0.88
QMEAN6 score	0.592	−1.90	0.606	−1.74	0.915	1.58	0.724	−0.43	0.678	−0.82	0.673	−1.04

Residues	788		703		321		683		914		742	
PDFs total energy	-		5790.3301		-		1348.0272		-		108,223	
DOPE score	-		−74,339.4609		-		−80,511.2891		-		−86,723.3	

**Table 3.** The conserved Ca^2+^-binding site in CAPN large subunit (DI-DIV) with protein structures.

PDB ID	Molecular description	The Ca^2+^ binding site	The residue conservation pattern *
1KXR	Ca^2+^ bound protease core of CAPN1(rat)	Site 1: Val99, Gly101, Asp106, Glu185 Site 2: Glu302, Asp309, Met 329, Asp331, Glu333	Site 1: **V**GDE Site 2: ED**M**DE Site 3: ADEE
1ZCM	CAPN1 protease core inhibited by ZLLYCH2F(human)	Site 1: Val99, Gly101, Asp106, Glu185 Site 2: Glu302, ASP309, Met329, Asp331, Glu333	
	Activated CAPN1 structure model (bovine)	Site 1: Val99, Asp100, Gly101, Thr103, Asp106, Glu185 Site 2: Glu302, ASP309, Met329, Asp331, Glu333 Site 3: Ala557, Asp560, Glu562, Glu567	
3BOW	Complex of CAPN2 and CAST(rat)	Site 1: Ile89, Gly90, Gly91, Asp96, Glu175 Site2: Glu292, Asp299, Gln319, Asp321, Glu323 Site3: Ala542, Asp545, Glu547, Glu552 Site 4: Asp585, Asp587, Ser589, Lys591, Glu596 Site 5: Asp615, Asp617, Ser618, Thr621, Glu626 Site 6: Asp658, Asn661	Site 1: **IG**GDE Site 2: EDQDE Site 3: ADEE Site 4: DD**S**LE Site 5:DD**RT**E Site 6:**D**DDN
3DF0	Complex of CAPN2 and CAST(rat)	Site 1: Ile89, Gly90, Gly91, Asp96, Glu175 Site2: Glu292, Asp299, Gln319, Asp321, Glu323 Site 3: Ala542, Asp545, Ala546, Glu547, Glu552 Site 4: Glu547, Asp585, Asp587, Ser589, Lys591, Glu596 Site 5: Asp615, Asp617, Ser618, Thr621, Glu626 Site 6: Asp570, Asp658, Asp660, Asn661	

* The presence of Ca^2+^ ions bound in the conservation patterns of CAPN large subunit with protein structure alignment in which residues are conserved between CAPN1 and CAPN2 represented by black, class-specific residues are indicated in blue and pink for CAPN1 and CAPN2, respectively.

**Table 4.** Identifying reference proteins for homology modeling the bovine CAPN1.

PDB ID	Molecular description	Experimental details	Length	Sequence identity (%)	Sequence similarity (%)	*E*-value	Template
1QXP:B	Like CAPN1 (rat)	X-ray Diffraction (2.80 Å)	788	74.6	82.4	0.0	T
1KXR:B	Ca^2+^ bound protease core of CAPN1 (rat)	X-ray Diffraction (2.07 Å)	321	80.2	85.8	1 × 10^−17^	T
3BOW:A	CAPN2 catalytic subunit (rat)	X-ray Diffraction (2.40 Å)	680	59.4	77	0.0	T
3BOW:C	CAST 4 domain (rat)	X-ray Diffraction (2.40 Å)	65	72.9	86.4	3 × 10^−10^	T
1KFX:L	Form I of CAPN2 (human)	X-ray Diffraction (3.15 Å)	640	57.1	75.7	0.0	-
1KFU:L	Form II of CAPN2 (human)	X-ray Diffraction (2.50 Å)	699	61.4	81.3	0.0	-
3DF0:A	CAPN2 catalytic subunit (rat)	X-ray Diffraction (2.95 Å)	676	59.5	77.5	0.0	-
1U5I:A	CAPN2 mutant Lys10Thr (rat)	X-ray Diffraction (2.86 Å)	625	56.1	73.4	0.0	-

**Table 5.** Key interaction contacts in the CAPN1/CAST4 complex.

The regulation system	Interaction regions	Calpain residues *
The catalytic subunit of CAPN1/subdomains AB of CAST4 complex from both bovines (model structure)	CAPN1 and CAST4 subdomain B	Leu73, Ser78, Lys79, **Ile83**, Cys108, Gln109, Gly110, Ala111, Leu112, Gly113, Cys115, Trp116, Lys171, **Lys174**, Leu175, **Val176**, His179, **Ser209**, Thr210, Glu212, Ser251, **Asp253**, **Ile254**, **Ser255**, **Ser256**, **Asp259**, **Met260**, **Ala262**, **Val263**, **Val269**, **Lys270**, Gly271, His272, Ala273, Trp298, **Glu300**, **Arg347**, Glu349, Cys384, Arg385, Asn386, Pro388, Trp392, Asp439, Met440, Thr442, Tyr448, **Lys463**, **Asp465**, Phe467, Leu468, **Ser469**, **Asn470**, **Ala471**, **Ser472**, **Arg475, Gln478**, Phe479, Ile480, Asn481, Leu482, Arg483, Phe503
	CAPN1 and CAST4 subdomain A	**Asn548**, Leu552, **Gln555, Leu556**, Ile571, **Arg574**, **Ile575**, **Lys578, His579**, **Asp581**, Phe612, Trp616, Ile619, **Arg620**, Leu623, Arg627, Asp630, **Leu631**, **Lys633**, Gly635

The overall CAPN2/CAST4 complex from both rats (PDB ID: 3BOW)	CAP2 and CAST4 subdomain B	Leu63, Gly100, Ala101, Leu102, Lys161, Leu169, Gly198, Ile244, Thr245, Asp249, Gly261, His262, Ala263, Trp288, Arg375, Asn376, Leu454, Thr455, Arg457, Ala458, Arg461, Phe465, Asn467, Phe489
	CAP2 and CAST4 subdomain A	Leu537, Gln540, Leu541, Ile556, Val560, Arg564, Trp601, Gln605, Gln608, Arg612, Asp615
	CAP2 and CAST4 subdomain C	Leu106, Leu110, Ile125, Val129, Arg132, His133, Trp170

* Interaction sites by residue character: class-specific (blue) and bovine only species-specific (red) trace residues.
